# Design and Synthesis of Cyclic ADP-4-Thioribose as a Stable Equivalent of Cyclic ADP-Ribose, a Calcium Ion-Mobilizing Second Messenger**

**DOI:** 10.1002/anie.201302098

**Published:** 2013-05-13

**Authors:** Takayoshi Tsuzuki, Natsumi Sakaguchi, Takashi Kudoh, Satoshi Takano, Masato Uehara, Takashi Murayama, Takashi Sakurai, Minako Hashii, Haruhiro Higashida, Karin Weber, Andreas H Guse, Tomoshi Kameda, Takatsugu Hirokawa, Yasuhiro Kumaki, Barry V L Potter, Hayato Fukuda, Mitsuhiro Arisawa, Satoshi Shuto

**Affiliations:** Faculty of Pharmaceutical Sciences, Hokkaido UniversityKita-ku, Sapporo 060-0812 (Japan); Faculty of Pharmaceutical Sciences and Center for Research and Education on Drug Discovery, Hokkaido UniversityKita-ku, Sapporo 060-0812 (Japan) E-mail: shu@pharm.hokudai.ac.jp; Department of Pharmacology, Juntendo University School of MedicineBunkyo-ku, Tokyo 113-8421 (Japan); Department of Biophysical Genetics, Kanazawa University Graduate School of MedicineKanazawa 920-8640 (Japan); University Medical Center Hamburg-Eppendorf, Center of Experimental Medicine, Department of Biochemistry and Signal TransductionMartinistr. 52, 20246 Hamburg (Germany); Computational Biology Research Center (CBRC), National Institute of Advanced Industrial Science and Technology (AIST)Koutou-ku, Tokyo 135-0064 (Japan); Faculty of Sciences, Hokkaido UniversityKita-ku, Sapporo 060-0810 (Japan); Department of Pharmacy and Pharmacology, University of BathBath BA2 7AY (UK)

**Keywords:** conformational analysis, nucleotides, second messengers, synthesis design, thioriboses

Cyclic ADP-ribose (cADPR, **1**, Scheme 1), originally isolated from sea urchins by Lee and co-workers,^1^ is a general mediator of intracellular Ca^2+^ ion signaling.^2^ Analogues of cADPR have been extensively designed and synthesized^3^, ^4^ because of their potential usefulness for investigating the mechanisms of cADPR-mediated Ca^2+^ release and application as lead structures for the development of drug candidates.^2^

**Scheme 1:**
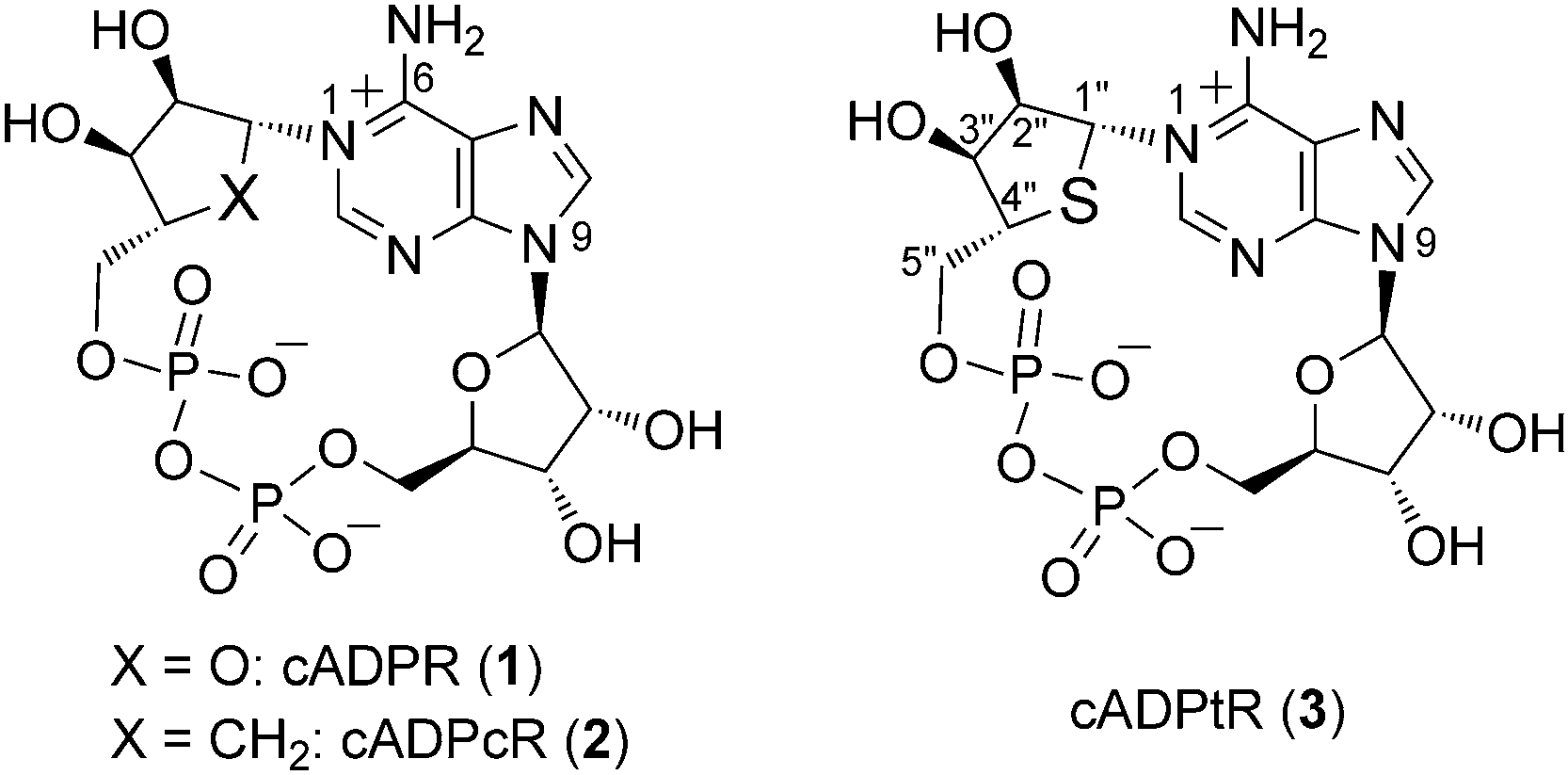
cADPR (**1**), cADPcR (**2**), and cADPtR (**3**).

cADPR is very unstable and can be hydrolyzed not only by cADPR hydrolase in cells but also in neutral aqueous solution at the labile N1-ribosyl linkage.^5^ We previously synthesized cyclic ADP-carbocyclic-ribose (cADPcR, **2**) as a stable mimic of cADPR, in which the oxygen in the N1-ribose ring of cADPR was replaced by methylene. cADPcR is both chemically and biologically stable and effectively mobilizes intracellular Ca^2+^ ions in sea urchin eggs and neuronal cells.4c However, cADPcR is almost inactive in T cells.4d

Although intensive studies of the signaling pathway that uses cADPR are still needed, its biological and chemical instability limits further studies of its physiological role. Therefore, stable analogues of cADPR mobilizing Ca^2+^ ions in various cells, including T cells, are needed. We designed a 4-thioribose analogue of cADPR, that is, cyclic ADP-4-thioribose (cADPtR, **3**), in which the N1-ribose of cADPR was replaced by a 4-thioribose. Herein, we describe the design, synthesis, biological effects, and conformational analysis of cADPtR as a stable equivalent of cADPR.

cADPR exists in an equilibrium between the *N*^6^-protonated amino form and the *N*^6^-deprotonated imino form (Scheme 2 a).^6^ The p*K*_a_ of cADPcR (8.9)4c is somewhat higher than that of cADPR (8.3).6a Thus, under physiological conditions, cADPR exists in a mixture of the protonated form and the deprotonated form, whereas cADPcR should be present mostly in the protonated form, which could affect its interaction with the target proteins.

**Scheme 2:**
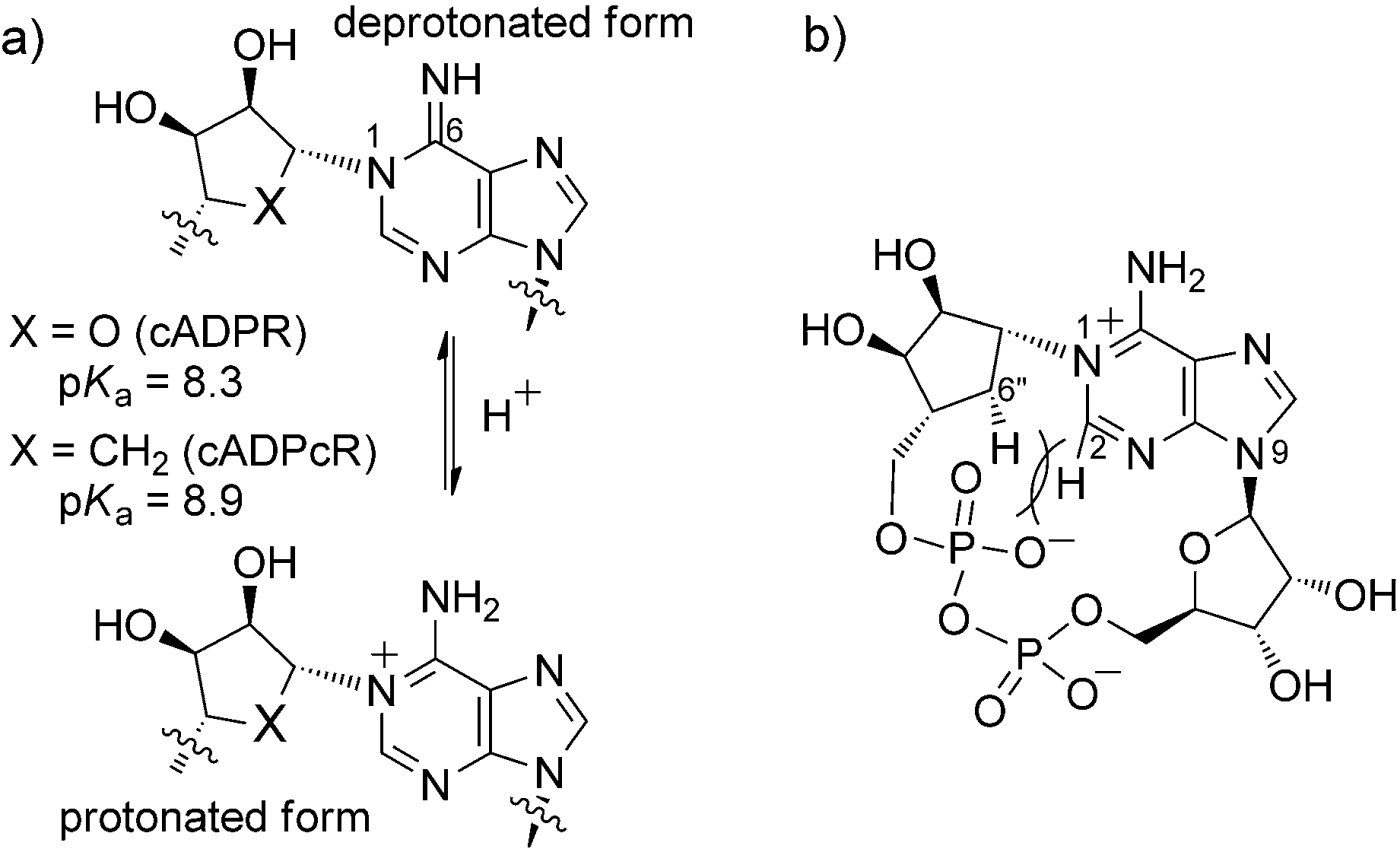
a) Equilibrium between the *N*^6^-protonated amino form and the *N*^6^-deprotonated imino form in cADPR and cADPcR. b) Possible steric repulsion between the H2 and the H6′′β in cADPcR.

In cADPR and its analogues, the most stable conformation is the one with minimal steric repulsion between the adenine moiety and both of the N1- and N9-ribose moieties. It should be noted that, in cADPcR, the H6′′β, which is absent in cADPR, is sterically repulsive to the adenine H2 (Scheme 2 b). Accordingly, the stable conformation of cADPcR might differ from that of cADPR owing to the steric effects, which might also affect its interaction with the target proteins.

We hypothesized that the above-mentioned p*K*_a_ value and conformational properties of cADPcR might explain its inactivity in T cells, and therefore designed cADPtR, because 4′-thionucleosides are useful bioisosteres of natural nucleosides,^7^ in which the *N*-4-thioriboyl linkage is more stable against both chemical and enzymatic hydrolysis than the *N*-ribosyl linkage of the natural nucleosides.^8^ Furthermore, the p*K*_a_ value of cADPtR should be similar to that of cADPR owing to the electron-withdrawing property of the sulfur atom.^9^ Also, the conformation of cADPtR, particularly, the spatial positioning of the N1-thioribose and adenine moieties, would be similar to that of cADPR because of the similar sp^3^ configuration of the oxygen and sulfur atoms. Thus, we predicted that cADPtR would be a stable cADPR equivalent.

In the synthesis of cADPtR (**3**), the key step was achieving stereoselective construction of the N1-β-thioribosyladenosine structure. Although no 1-amino-4-thioribose derivatives such as **4** have been reported to date, **4** is likely to be present as an equilibrated anomeric mixture **4** α and **4** β (Scheme 3) owing to the electron-donating property of the hemiaminal ether nitrogen at the 1-position. We speculated that stereoselective construction of the N1-β-thioribosyladenosine structure could be achieved, because the α-face of **4** would be more sterically hindered than the β-face owing to its 5,5-*cis* ring system, so that the β-anomer **4** β might preferentially react with a nucleoside derivative **5**.^10^ Thus, over the course of the reaction, the relatively less reactive α-anomer **4** α would not undergo the condensation reaction, but rather would be converted into the more reactive **4** β through the equilibrium reaction, which would lead to an accumulation of the desired β-product **6** β (Scheme 3).

**Scheme 3:**
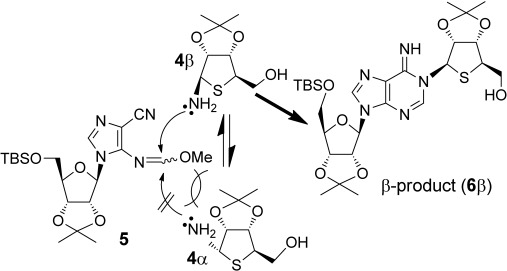
Hypothesis for the stereoselective formation of the β-product **6** β by way of an α/β equilibrium.

The synthesis of **4** is shown in Scheme 4. Oxidation of **7**^11^ with a subsequent Pummerer rearrangement afforded the 1-acetoxy product **9** (α/β=1:5). Treatment of **9** with TMSN_3_/SnCl_4_ gave the β-azide **10** stereoselectively, probably because of the steric demand of the reaction intermediate. Reduction of the azido group of **10**, followed by deprotection of the *O*-acetyl group gave **4**, which was an anomeric mixture (α/β=1:2) as expected.

**Scheme 4:**
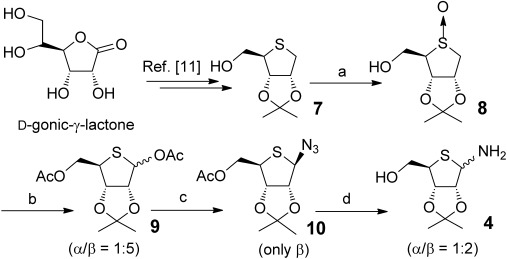
Synthesis of the 4-thioribosylamine **4**. a) *m*CPBA, CH_2_Cl_2_, −78 °C, 91 %; b) Ac_2_O, 100 °C, 64 %; c) TMSN_3_, SnCl_4_, CH_2_Cl_2_, 0 °C, 86 %; d) 1) H_2_, Pd-C, MeOH, 2) MeOH, reflux, quant. *m*CPBA=*meta*-chloroperoxybenzoic acid; TMSN_3_=trimethylsilyl azide.

The key step, the condensation between **4** and **5**, was then examined. We found that treatment of **4** with **5** (2.1 equiv) in MeOH at room temperature produced the β-product **6** β in 61 % yield, along with 5 % of the α-product **6** α,^12^ where **4** was recovered in 17 % yield (Scheme 5). Thus, the desired β-product **6** β was successfully obtained in 73 % conversion yield from **4**, probably owing to the α/β-equilibrium between **4** α and **4** β.

**Scheme 5:**
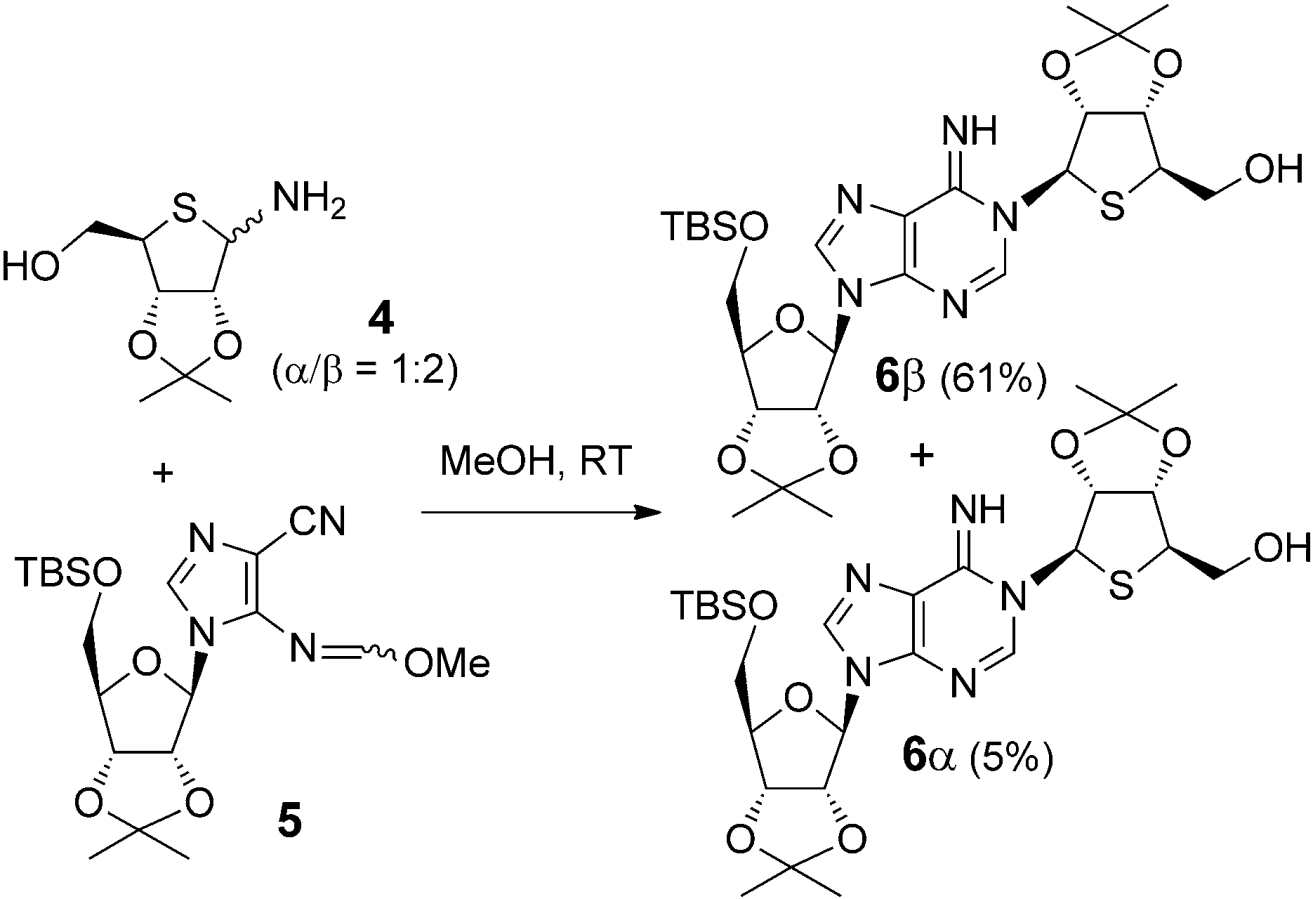
Stereoselective condensation giving the β-product **6** β.

Synthesis of cADPtR was investigated next (Scheme 6). After protecting group manipulation of **6** β, treatment of the resulting **12** with *S*,*S*′-diphenylphosphorodithioate (PSS)/2,4,6-triisopropylbenzenesulfonyl chloride (TPSCl) in pyridine,^13^ followed by removal of the 5′′-*O*-DMTr group gave the 5′-bis-*S*-(phenyl)phosphorothioate **14**. Phosphorylation of **14** by the normal Yoshikawa method with POCl_3_ was unsuccessful.^14^ However, treatment of **14** with a zwitterionic phosphorylating reagent **15** [15] in pyridine at −30 °C led to the corresponding phosphorylation product (detected by HPLC analysis), which was further treated with H_3_PO_2_ and Et_3_N in pyridine^16^ to afford the phosphorylated **16**.

**Scheme 6:**
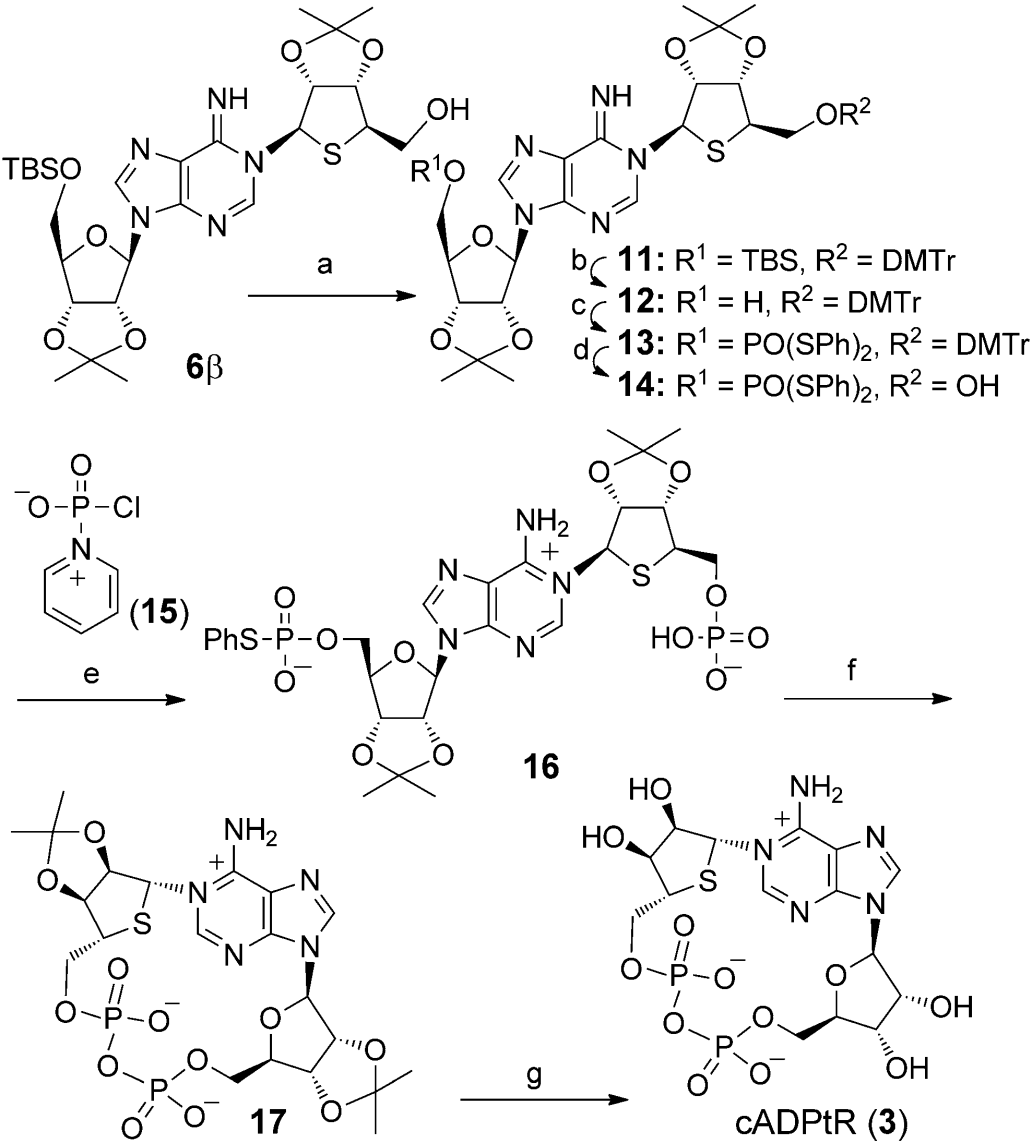
Synthesis of cADPtR (**3**). a) DMTrCl, pyridine, 81 %; b) TBAF, AcOH, THF, quant; c) PSS, TPSCl, pyridine, −15 °C, 72 %; d) aq. AcOH, 90 %; e) 1) **15**, pyridine, −30 °C, then TEAA, 2) H_3_PO_2_, Et_3_N, pyridine, 0 °C, 46 %; f) AgNO_3_, Et_3_N, 3 Å molecular sieves, pyridine, 76 %; g) aq. HCO_2_H, 49 %. DMTrCl=dimethoxytrityl chloride; TBAF=tetra-*n*-butylammonium fluoride; PSS=*S*,*S*′-diphenylphosphorodithioate; TPSCl=2,4,6-triisopropylbenzenesulfonyl chloride; TEAA=triethylammonium acetate buffer

Cyclization of the 18-membered pyrophosphate ring was achieved using the phosphorothioate **16** as a substrate, by the Ag^+^ promoted intramolecular condensation that we developed previously.4b,c Thus, when a solution of **16** in pyridine was slowly added to a mixture of a large excess of AgNO_3_ and Et_3_N in the presence of 3 Å molecular sieves in pyridine at room temperature,4b,c, 13 the desired product **17** was obtained in 76 % yield. Finally, removal of the isopropylidene groups of **17** produced the target cADPtR.

The p*K*_a_ value of cADPtR (**3**) was determined based on the pH-dependent UV spectral change owing to protonation/deprotonation at the *N*^6^ position of the adenine ring. Thus, the p*K*_a_ of cADPtR was determined to be 8.0, which is similar to that of cADPR (p*K*_a_=8.3)6a and about one pH unit lower than that of cADPcR (pK_a_=8.9).4c

Structures of cADPR (**1**), cADPcR (**2**), and cADPtR (**3**) were constructed from molecular dynamics calculations using a simulated annealing method based on the NOE constraints of the intramolecular proton pairs measured in D_2_O (for details, see Supporting Information), which are shown in Figure 1 a–c. To clarify the structural differences in detail, the three obtained structures were superimposed (Figure 1 d), revealing that the cADPtR structure (red) resembles that of cADPR (blue). The cADPcR structure (green), however, is not similar to those of the other two compounds, and the relative special arrangement of the N1-carbocyclic ribose and the adenine of cADPcR clearly differs from those of the other two compounds, as expected. The distances between the 6′′C and the adenine H2 of cADPtR (3.6 Å) is significantly longer than the corresponding distances of cADPR (2.3 Å) and cADPtR (2.5 Å). To confirm the validity of the obtained structures, the cADPR structure solved by X-ray crystallographic analysis (white)2c, 6a was superimposed onto the three calculated structures (Figure 1 e). This crystal cADPR structure resembles the calculated cADPR and cADPtR structures, which suggests our computational structure determination was appropriate. Therefore, the p*K*_a_ and conformational properties of cADPtR precisely mimic those of cADPR.

**Figure 1:**
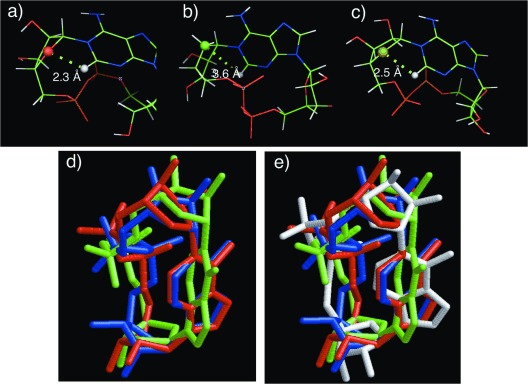
Structures of a) cADPR, b) cADPcR, c) cADPtR from molecular dynamics calculations with a simulated annealing method using the NOE data in D_2_O; adenine H2 (white sphere), O4′′ in cADPR (red sphere), C6′′ in cADPcR (green sphere) and S4′′ in cADPtR (yellow sphere). d) Superimposed displays of the calculated structures; cADPR (blue), cADPcR (green), cADPtR (red). e) The crystal structure of cADPR (white) was also superimposed onto the three structures.

The biological stability of cADPtR (**3**) was investigated with a rat brain microsomal extract that contained cADPR degradation enzymes.^5^ cADPtR was completely resistant to degradation in the extract, whereas cADPR was rapidly degraded (Figure 2).

**Figure 2:**
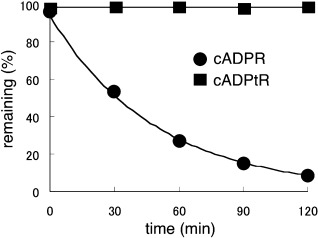
Stability of cADPtR in rat brain microsomal extract.

We tested the Ca^2+^ ion-mobilizing ability of cADPtR (**3**), cADPR (**1**), and cADPcR (**2**) with a sea urchin egg homogenate^17^ (Figure 3). cADPR and cADPcR induced the release of Ca^2+^ ions in a concentration-dependent manner with an EC_50_ value of 214 nm and 54 nm, respectively. cADPtR was highly active (EC_50_=36 nm), and was about sixfold more potent than cADPR and even more potent than cADPcR.

**Figure 3:**
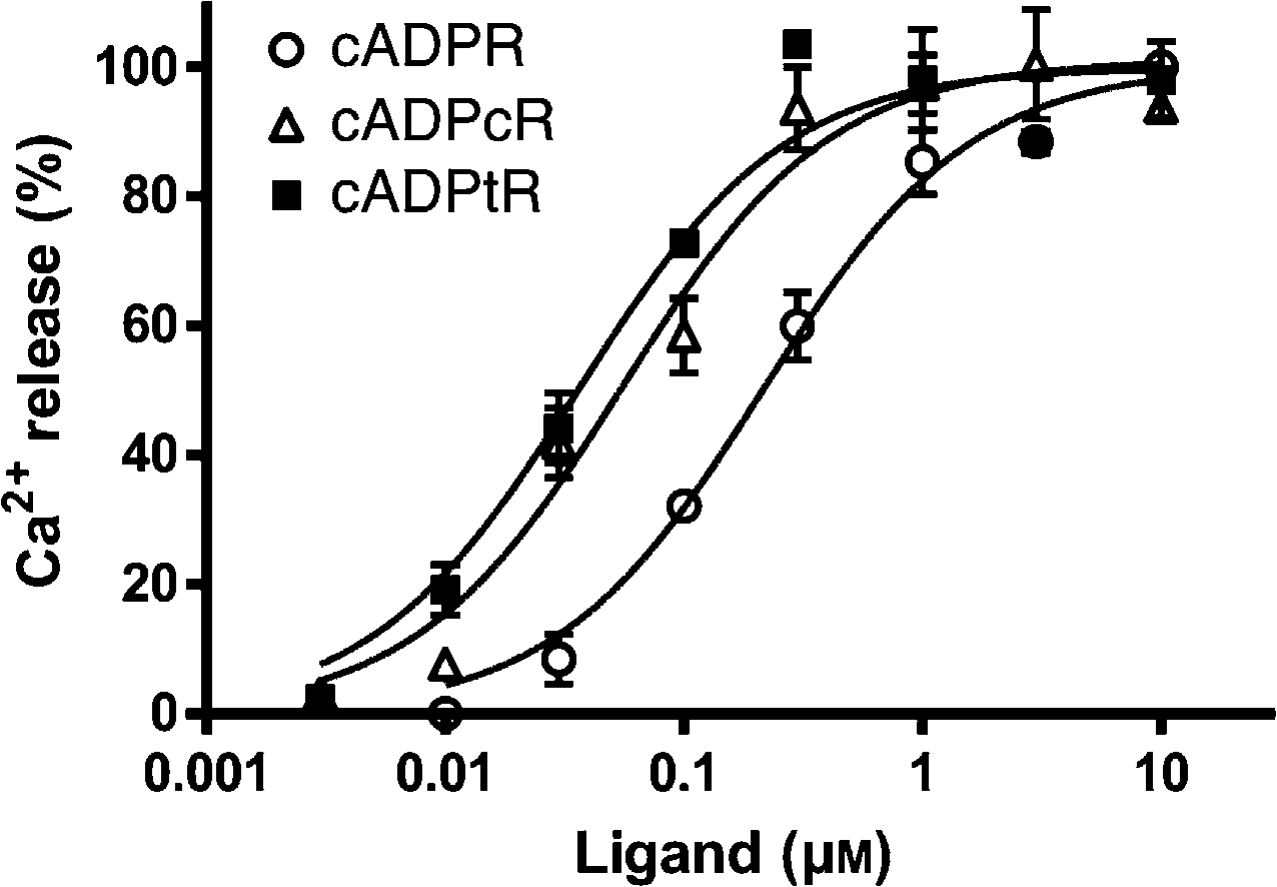
Ca^2+^ ion-mobilizing activity of cADPR, cADPcR, and cADPtR in sea urchin egg homogenate. Data are the mean±SEM of 3–6 experiments.

The effect of cADPtR (**3**) on cytosolic Ca^2+^ ion mobilization in NG108-15 neuronal cells was tested.^18^ Application of 100 μm cADPtR induced persistent increases in the Ca^2+^ level within the cells: the mean Ca^2+^ ion level measured four minutes after application of cADPtR was 116±2.3 % of the resting level (mean±SEM, *n*=6). The amplitude produced by cADPtR addition was equivalent to or significantly greater than that induced by cADPR (Figure S3).

The Ca^2+^ ion-mobilizing effect of cADPtR (**3**) was evaluated using saponin-permeabilized Jurkat T cells.^19^ Both cADPtR and cADPR (**1**) evoked rapid Ca^2+^ ion release upon addition to the permeabilized cell suspension indicating that they induce similar mechanisms of Ca^2+^ release (Figure 4 a). cADPR and cADPtR had very similar concentration-response curves (Figure 4 b). Our previous work revealed that cADPcR shifted its Ca^2+^ ion-mobilizing activity to much higher concentrations.4d, 19b In contrast, cADPtR was almost as active as cADPR. The structural and electrostatic features of cADPtR, analogous to cADPR, would make it as biologically active as cADPR in various systems including T cells, although the target proteins of cADPR in these systems are thought to be different.4d

**Figure 4:**
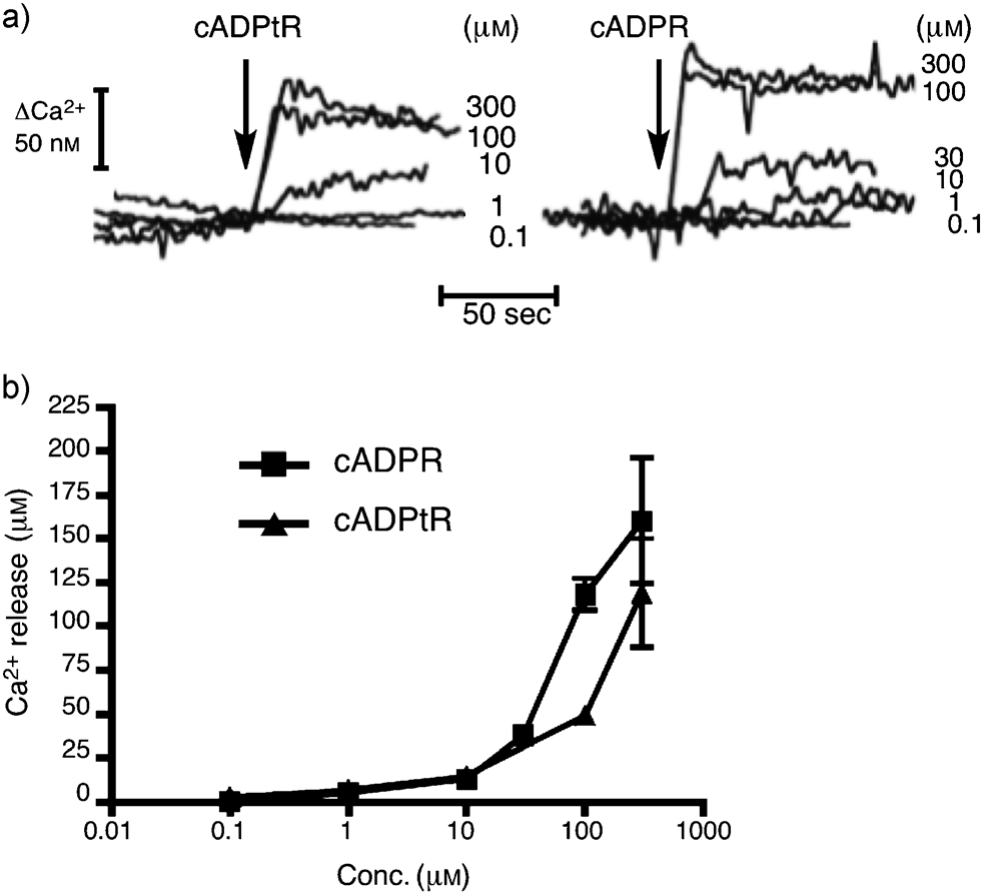
Effect of cADPR and cADPtR on Ca^2+^ ion signaling in permeabilized Jurkat T cells. a) Representative traces. b) Data presented as the mean±SEM (*n*=2–8).

In summary, we have synthesized cADPtR and demonstrated that it is stable and functions similar to cADPR in various biological systems. Because of its stability and high potency, cADPtR should be an effective biological tool as the first stable equivalent of cADPR.

## Supplementary material

As a service to our authors and readers, this journal provides supporting information supplied by the authors. Such materials are peer reviewed and may be re-organized for online delivery, but are not copy-edited or typeset. Technical support issues arising from supporting information (other than missing files) should be addressed to the authors.

## References

[1] Clapper DL, Walseth TF, Dargie PJ, Lee HC (1987). J. Biol. Chem.

[2a] Galione A (1993). Science.

[2b] Guse AH (1999). Cell. Signalling.

[2c] Lee HC (2002). Cyclic ADP-ribose and NAADP: Structures, Metabolism and Functions.

[2d] Lee HC (2012). J. Biol. Chem.

[3a] Lee HC, Aarhus R, Walseth TH (1993). Science.

[3b] Moreau C, Kirchberger T, Zhang B, Thomas MP, Weber K, Guse AH, Potter BVL (2012). J. Med. Chem.

[4a] Shuto S, Shirato M, Sumita Y, Ueno Y, Matsuda A (1998). J. Org. Chem.

[4b] Fukuoka M, Shuto S, Minakawa N, Ueno Y, Matsuda A (2000). J. Org. Chem.

[4c] Shuto S, Fukuoka M, Manikowsky M, Ueno Y, Nakano T, Kuroda R, Kuroda H, Matsuda A (2001). J. Am. Chem. Soc.

[4d] Kudoh T, Fukuoka M, Ichikawa S, Murayama T, Ogawa Y, Hashii M, Higashida H, Kunerth S, Weber K, Guse AH, Potter BVL, Matsuda A, Shuto S (2005). J. Am. Chem. Soc.

[4e] Swarbrick JM, Potter BVL (2012). J. Org. Chem.

[4f] Yu PL, Zhang AH, Hao BX, Zhao YJ, Zhang LH, Lee HC, Zhang L, Yue J (2012). J. Biol. Chem.

[5] Lee HC, Aarhus R (1993). Biochim. Biophys. Acta Protein Struct. Mol. Enzymol.

[6a] Kim H, Jacobson EL, Jacobson MK (1993). Biochem. Biophys. Res. Commun.

[6b] Lee HC, Aarhus R, Levitt D (1994). Nat. Struct. Biol.

[7a] Reist EJ, Gueffroy DE, Goodman L (1964). J. Am. Chem. Soc.

[7b] Dyson MR, Coe PL, Walker RT (1991). J. Med. Chem.

[7c] Bellon L, Barascut JL, Maury G, Divira G, Goody R, Imbach JL (1993). Nucleic Acids Res.

[7d] Naka T, Minakawa N, Abe H, Kaga D, Matsuda A (2000). J. Am. Chem. Soc.

[8a] Elzagheid ML, Oivanen M, Walker RT, Secrist JA (1999). Nucleosides Nucleotides Nucleic Acids.

[8b] Toyohara J, Gogami A, Hayashi A, Yonekura Y, Fujibayashi Y (2003). J. Nucl. Med.

[9a] Ganellin CR, Owen DAA (1977). Agents Actions.

[9b] Salzner U, Schleyer PVR (1993). J. Am. Chem. Soc.

[10] Hutchinson EJ, Taylor BF, Blackburn GM (1997). J. Chem. Soc. Chem. Commun.

[11] Jeong LS, Lee HW, Jacobson KA, Kim HO, Shin DH, Lee JA, Gao Z-G, Lu C, Duong HT, Gunaga P, Lee SK, Jin DZ, Chun MW, Moon HW (2006). J. Med. Chem.

[13] Sekine M, Hata T (1993). Curr. Org. Chem.

[14] Yoshikawa M, Kato T, Takenishi T (1969). Bull. Chem. Soc. Jpn.

[15] Asseline U, Thuong NT (1988). Nucleosides Nucleotides.

[16] Hata T, Kamimura T, Urakami K, Kohno K, Sekine M, Kumagai J, Shinozaki K, Miura K (1987). Chem. Lett.

[17] Shiwa M, Murayama T, Ogawa Y (2002). Am. J. Physiol. Regul. Integr. Comp. Physiol.

[18] Amina S, Hashii M, Ma WJ, Yokoyama S, Lopatina O, Liu HX, Islam MS, Higashida H (2010). J. Neuroendocrinol.

[19a] Schwarzmann N, Kunerth S, Weber K, Mayer GW, Guse AH (2002). J. Biol. Chem.

[19b] Guse AH, Cakir-Kiefer C, Fukuoka M, Shuto S, Weber K, Matsuda A, Mayer GW, Oppenheimer N, Schuber F, Potter BVL (2002). Biochemistry.

