# Supplementary material for: Design and Synthesis of Cyclic ADP-4-Thioribose as a Stable Equivalent of Cyclic ADP-Ribose, a Calcium Ion-Mobilizing Second Messenger
Source: Angew Chem Int Ed Engl. 2013 May 13;52(26):6633–7. doi: 10.1002/anie.201302098 (PMC3738939; doi:10.1002/anie.201302098)
Supplement: Supplementary file 1 [file anie0052-6633-SD1.pdf]

Angewandte Chemie
Eine Zeitschrift der Gesellschaft Deutscher Chemiker

Supporting Information
© Wiley-VCH 2013
69451 Weinheim, Germany

Design and Synthesis of Cyclic ADP-4-Thioribose as a Stable Equivalent of Cyclic ADP-Ribose, a Calcium Ion-Mobilizing Second Messenger**

*Takayoshi Tsuzuki, Natsumi Sakaguchi, Takashi Kudoh, Satoshi Takano, Masato Uehara, Takashi Murayama, Takashi Sakurai, Minako Hashii, Haruhiro Higashida, Karin Weber, Andreas H. Guse, Tomoshi Kameda, Takatsugu Hirokawa, Yasuhiro Kumaki, Barry V. L. Potter, Hayato Fukuda, Mitsuhiro Arisawa, and Satoshi Shuto**

anie_201302098_sm_miscellaneous_information.pdf

Supporting Information Available

Contents: Detail procedures for the synthesis of compounds**,** biological evaluations and computations. NOE and HMBC data of **6β** and **6α** **(**Figure S1**)**, important correlations in NOESY spectra of cADPtR (Figure S2), effects of cADPtR on Ca^{2+} increases in permeabilized NG108-15 cells (Figure S3), and ^{1}H NMR charts of compounds.

1,4-Dideoxy-1,4-episulfinyl-2,3-*O*-isopropylidene-D-ribitol (8). To a solution of **7** (1.90 g, 10.0 mmol) in CH_2Cl_2 (30 mL) was added slowly a solution of *m*CPBA (3.18g, 12.0 mmol) in CH_2Cl_2 (60 mL) at -78 °C, and the mixture was stirred at the same temperature for 20 min. To the mixture was added aqueous $Na_2S_2O_3$, and the resulting mixture was partitioned between EtOAc and H_2O, and the organic layer was washed with aqueous saturated $NaHCO_3$ and brine, dried (Na_2SO_4), and evaporated. The residue was purified by column chromatography (SiO_2, hexane/AcOEt = 3/1 then 1/1) to give **8** (1.88 g, 91%, white amorphous solid): ^{1}H-NMR of one isomer (500 MHz, $CDCl_3$) δ 1.33 (3H, s, isopropylidene-CH_3), 1.47 (3H, s, isopropylidene-CH_3), 3.29 (1H, dd, *J* = 14.3, 6.3 Hz, H-1), 3.42 (1H, ddd, *J* = 5.7, 5.7, 2.9 Hz, H-4), 3.45 (1H, dd, *J* = 14.3, 5.7 Hz, H-1) 4.10 (1H, dd, *J* = 12.6, 5.7 Hz, H-5), 4.34 (1H, dd, *J* = 12.6, 2.9 Hz, H-5), 4.80 (1H, br, OH), 5.10 (1H, dd, *J* = 5.7, 3.4 Hz, H-3), 5.20 (1H, ddd, *J* = 6.3, 5.7, 3.4 Hz, H-2); ^{13}C-NMR (125 MHz, $CDCl_3$) δ 24.61, 27.12, 57.06, 57.06, 58.11, 65.63, 79.76, 82.22, 112.11; HR-MS (EI) calcd for $C_8H_{14}O_4S_2$ 206. 06128 (M^+), found 206. 06169

1,5-*O*-Diacetyl-2,3-*O*-isopropylidene-4-thio-D-ribose (9). A solution of **8** (135 mg, 0.44 mmol) in Ac_2O (3 mL) was heated at 100 °C for 28 h, and then evaporated. The residue was partitioned between EtOAc and H_2O, and the organic layer was washed with aqueous saturated $NaHCO_3$ and brine. The aqueous layers were combined and extracted with $CHCl_3$. The organic layers were combined, dried (Na_2SO_4), and evaporated. The residue was purified by column chromatography (SiO_2, hexane/AcOEt = 6/1, 3/1 then 1/1) to give **9** (93 mg, 64%, *J* = 11.5, 5.7 Hz, H-5), 4.89-4.91 (2H, m, H-2, H-3), 6.04 (1H, s, H-1), for α-anomer, δ 1.36 (3H, s, isopropylidene-CH_3), 1.54 (3H, s, isopropylidene-CH_3), 2.10 (3H, s, Ac), 2.15 (3H, s, Ac), 3.90 (1H, m, H-4), 4.19 (1H, dd, *J* = 11.5, 6.3 Hz, H-5), 4.35 (*J* = 11.5, 5.7 Hz, H-5), 4.89-4.91 (2H, m, H-2, H-3), 6.04 (1H, s, H-1), for α-anomer, δ 1.36 (3H, s, isopropylidene-CH_3), 1.54 (3H, s, isopropylidene-CH_3), 2.10 (3H, s, Ac), 2.15 (3H, s, Ac), 3.90 (1H, m, H-4), 4.19 (1H, dd, *J* = 11.5, 6.3 Hz, H-5), 4.35 (1H, dd, *J* = 11.5, 6.3 Hz, H-5), 4.67 (1H, dd, *J* = 6.3, 4.0 Hz, H-3), 4.88 (1H, m, H-2), 6.09 (1H, d, *J* = 5.2 Hz, H-1) ^{13}C-NMR (100 MHz, $CDCl_3$) δ 20.85, 21.21, 24.61, 26.38, 53.85, 65.71, 85.13, 87.17, 88.54, 111.29, 169.16, 170.49; HR-MS (FAB, positive) calcd for $C_{12}H_{18}NaO_6S$ 313.0722 [$(M+Na)^+$], found 313.0717.

5-*O*-Acetyl-2,3-*O*-isopropylidene-4-thio-β-D-ribofuranosylazide (10). To a solution of **9** (2.26g, 7.78 mmol) and $TMSN_3$ (3.09 mL, 23.3 mmol) in CH_2Cl_2 (20 mL) was added a solution of $SnCl_4$ (223 mL, 1.91 mmol) in CH_2Cl_2 (10 mL) at 0 °C, and the mixture was stirred at the same temperature for 5 min. To the mixture was added aqueous saturated $NaHCO_3$, and the resulting white precipitate was filtered off with Celite. The filtrate was washed with aqueous saturated $NaHCO_3$ and brine, dried (Na_2SO_4), and evaporated. The residue was purified by column chromatography (SiO_2, hexane/AcOEt = 20/1) to give **10** (1.84g, 86%, yellow oil): ^{1}H-NMR (400 MHz, $CDCl_3$) δ 1.31 (3H, s, isopropylidene-CH_3), 1.50 (3H, s, isopropylidene- CH_3), 2.11 (3H, s, Ac), 3.63 (1H, dd, *J* = 10.0, 5.4 Hz, H-4), 4.12 (1H, dd, *J* = 11.3, 10.0 Hz, H-5), 4.27 (1H, dd, *J* = 11.3, 5.4 Hz, H-5), 4.64 (1H, d, *J* = 5.4 Hz, H-3), 4.86 (1H, d, *J* = 5.4 Hz, H-2), 5.18 (1H, s, H-1); ^{13}C-NMR (100 MHz, $CDCl_3$) δ 20.73,

24.51, 26.26, 54.29, 65.21, 76.13, 85.59 89.06, 111.23, 170.40; HR-MS (FAB, positive) calcd for $C_{10}H_{15}N_3NaO_4S$ 296.0681 [$(M+H)^+$], found 296.0695.

2,3-*O*-Isopropylidene-4-thio-D-ribofuranosylamine (4). A mixture of **10** (1.25 g, 4.57 mmol) and Pd/C (10%, 630 mg) in MeOH (45 mL) was stirred under atmospheric pressure of H_2 at room temperature for 1 h, and then the catalysts were filtered off with Celite. The filtrate was evaporated, and the residue was purified by column chromatography (NH-silica gel, hexane/AcOEt = 6/1, 2/1 then 1/3) to give **4** (1.09 g, quant., α/β = 1/2, brown oil): ^{1}H-NMR (500 MHz, $CDCl_3$) δ 1.30 (2H, s), 1.36 (1H, s), 1.52, (2H, s), 1.57 (1H, s), 1.83 (2H, brs), 3.58 (2/3H, dd, *J* = 7.7, 5.4 Hz), 3.73 (1/3H, ddd, *J* = 6.3, 5.9 Hz), 4.32 (2/3H, dd, *J* = 11.7, 7.7 Hz), 4.43 (2/31H, dd, *J* = 11.7, 5.4 Hz), 4.49 (1/3H, dd, *J* = 11.3, 5.9 Hz), 4.60-4.66 (2/3H, m), 4.71-4.74 (1H, m), 4.79 (2/3H, d, *J* = 5.2 Hz, b-H-1), 4.92 (1/3H, dd, *J* = 4.5 Hz), 5.04 (2/3H, d, *J* = 4.5 Hz); ^{13}C-NMR (100 MHz, $CDCl_3$) δ -5.64, -5.51, 18.23, 24.36, 25.27, 25.80, 26.50, 27.22, 57.20, 63.68, 64.31, 64.63, 81.06, 85.99, 86.45, 87.57, 91.20, 91.65, 95.13, 110.33, 113.74, 117.26, 132.20, 148.52, 151.04; HR-MS (EI) calcd for $C_8H_{15}NO_3S$ 205.0773 (M^+), found 205.0773.

***N*1-(2,3-*O*-Isopropylidene-4-thio-β-D-ribofuranosyl)-5'-*O*-(*tert*-butyldimethylsilyl)-2',3'-*O*-isopropylideneadenosine (6β).** A solution of **4** (381 mg, 1.87 mmol) and **5** (1.79g, 4.00 mmol) in MeOH (10 mL) was stirred at room temperature for 10 h, and then evaporated. The residue was purified by flash column chromatography (silica gel, hexane/AcOEt = 1/2, AcOEt , then AcOEt/MeOH = 9/1) to give **6β** (700 mg, 61%, white amorphous solid) and **5α** (59 mg, 5%, white amorphous solid). **6β**: ^{1}H-NMR (500 MHz, CDCl) δ 0.04 (3H, s, Si-CH_3), 0.05 (3H, s, Si-CH_3), 0.86 (9H, s, *tert*-butyl), 1.35 (3H, s, isopropylidene-CH_3), 1.39 (3H, s, isopropylidene-CH_3), 1.61 (3H, s, isopropylidene-CH_3), 1.63 (3H, s, isopropylidene-CH_3), 3.77 (1H, dd, *J* = 11.4, 4.0, 4.0 Hz, H-5'), 3.83-3.88 (2H, m, H-5', H-5"), 3.90 (1H, m, H-4"), 4.01 (1H,dd, *J* = 11.4, 3.7 Hz, H-5"), 4.39 (1H, m, H-4'), 4.88 (1H, dd, *J* = 6.3, 3.4 Hz, H-3'), 4.99 (1H, dd, *J* = 5.2, 1.7 Hz, H-3"), 5.06 (1H, dd, *J* = 6.3, 2.3 Hz, H-2'), 5.51 (1H, dd, *J* = 5.2, 3.4 Hz, H-2"), 6.00 (1H, d, *J* = 3.4 Hz, H-1"), 6.02 (1H, d, *J* = 2.3 Hz, H-1'), 7.85 (1H, s, H-8), 7.88 (1H, s, H-2); ^{13}C-NMR (125 MHz, $CDCl_3$) δ -5.58, -5.47, 18.25 25.25, 25.80, 27.13, 27.92, 55.53, 63.37, 64.75, 74.54, 81.19, 85.23, 85.75, 86.22, 86.95, 91.15, 111.66, 114.09, 123.75, 137.20, 140.30, 146.81, 153.31; UV (MeOH) λ_{max} = 260 nm; LR-MS (FAB, positive) m/z 610 [$(M+H)^+$]; Anal. Calcd for $C_{27}H_{43}N_5O_7SSi$: C, 53.18; H, 7.11, N, 11.48. Found C, 52.88; H, 6.95; N, 11.35; NOE irradiated H-2/ observed H-2" (3.8%), irradiated H-1"/ observed H-4" (2.3%), irradiated H-2"/ observed H-2 (4.7%), irradiated H-4"/ observed H-1" (3.2%). **6α**: ^{1}H-NMR (500 MHz, CDCl) δ 0.05 (3H, s, Si-CH_3), 0.05 (3H, s, Si-CH_3), 0.87 (9H, s, *tert*-butyl), 1.35 (3H, s, isopropylidene-CH_3), 1.39 (3H, s, isopropylidene-CH_3), 1.61 (3H, s, isopropylidene-CH_3), 1.63 (3H, s, isopropylidene-CH_3), 3.56 (1H, d, *J* = 4.1, .4.1 Hz, H-4"), 3.76 (1H, dd, *J* = 11.3, 3.6 Hz, H-5'), 3.84-3.88 (2H, m, H-5', H-5"), 3.93 (1H, dd, *J* = 10.9, 4.1 Hz, H-5"), 4.39 (1H, ddd, *J* = 6.3, 3.6, 3.6 Hz, H-4'), 4.90 (1H, dd, *J* = 6.3, 2.7 Hz, H-3'), 4.95 (1H, d, *J* = 5.9 Hz, H-3"), 5.01 (1H, dd, *J* = 5.9,

4.5 Hz, H-2”), 5.09 (1H, dd, *J* = 6.3, 2.7 Hz, H-2’), 6.05 (1H, d, *J* = 2.7 Hz, H-1’), 7.02 (1H, d, *J* = 4.5 Hz, H-1”), 7.83 (1H, s, H-8), 8.42 (1H, s, H-2); ^{13}C-NMR (125 MHz, $CDCl_3$) δ -5.54, -5.44, 18.29, 24.22, 25.33, 25.83, 25.97, 27.16, 54.36, 61.15, 63.34, 65.17, 80.59, 81.19, 85.30, 85.87, 86.99, 91.16, 111.38, 114.09, 122.77, 136.83, 140.88, 147.76, 155.00; UV (MeOH) λ_{max} = 259 nm; HR-MS (FAB, positive) calcd for $C_{28}H_{43}N_5O_7SSi$ 610.2731 (MH^+), found 610.2727. NOE irradiated H-2/ observed H-4” (1.0%), irradiated H-1”/ observed H-2” (10.7%), irradiated H-2”/ observed H-1” (8.4%), irradiated H-4”/ observed H-2 (1.7%).

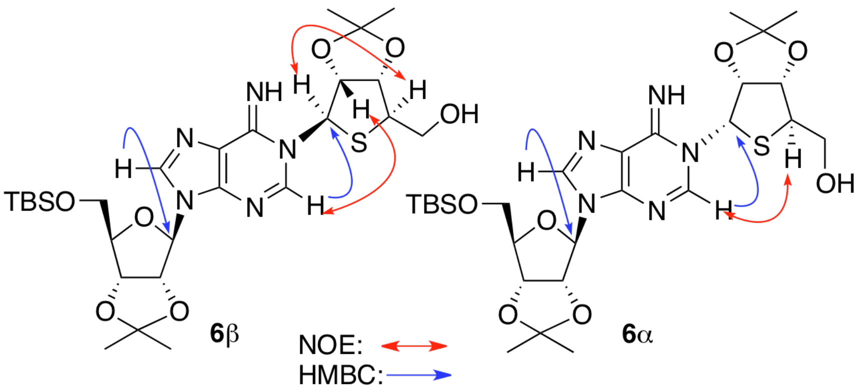

Figure S1. NOE and HMBC data of **6β** and **6α**.

***N*1-(2,3-*O*-Isopropylidene-4-thio-5-*O*-dimethoxytrityl-β-D-ribofuranosyl)-5’-*O*-(*tert*-butyldimethylsilyl)-2’,3’-*O*-isopropylideneadenosine (11).** A solution of **6β** (889 mg, 1.46 mmol) and DMTrCl (989 mg, 2.92mmol) in pyridine (5 mL) was stirred at room temperature for 12 h. After addition of MeOH, the resulting mixture was partitioned between EtOAc and 1 M aqueous HCl, and the organic layer was washed with H_2O and brine, dried (Na_2SO_4), and evaporated. The residue was purified by column chromatography (silica gel, hexane/AcOEt = 6/1, 3/1, 3/2, and 2/3) to give **11** (1.08 g, 81%, white amorphous solid): ^{1}H-NMR (500 MHz, $CDCl_3$) δ 0.05 (3H, s, Si-CH_3), 0.06 (3H, s, Si-CH_3), 0.88 (9H, s, *tert*-butyl), 1.30 (3H, s, isopropylidene-CH_3), 1.38 (3H, s, isopropylidene-CH_3), 1.59 (3H, s, isopropylidene-CH_3), 1.62 (3H, s, isopropylidene-CH_3), 3.40-3.46 (2H, m, H-5”×2), 3.77 (1H, m, H-5’), 3.78 (3H, s, OCH_3), 3.78 (3H, s, OCH_3), 3.80-3.85 (2H, m, H-4”, H-5’), 4.38 (1H, ddd, *J* = 5.7, 4.0, 4.0 Hz, H-4’), 4.72 (1H, dd, *J* = 5.7, 4.0 Hz, H-3”), 4.86 (1H, dd, *J* = 6.3, 5.7 Hz, H-3’), 4.89 (1H, dd, *J* = 5.7, 2.3 Hz, H-2”), 4.97 (1H, dd, *J* = 6.3, 2.9 Hz, H-2’) 6.02 (1H, d, *J* = 2.9 Hz, H-1’), 6.52 (1H, d, *J* = 2.3 Hz, H-1”), 6.81-7.47 (13H, m, Ar), 7.82 (1H, s, H-8), 8.12 (1H, s, H-2); ^{13}C-NMR (125 MHz, $CDCl_3$) δ -5.58, -5.47, 18.22, 25.21, 25.28, 25.79, 27.13, 27.35, 54.95, 55.05, 63.30, 64.85, 66.78, 81.06, 84.82, 85.25, 86.53, 86.79, 89.22, 90.86, 112.38, 113.03, 113.05, 114.01, 123.23, 126.66, 127.73, 127.95, 129.92, 129.96, 135.46, 135.60, 136.51, 140.35, 144.47, 145.55, 153.98, 158. 37; LR-MS (FAB, positive) m/z 882 [$(M+H)^+$]; UV (MeOH) λ_{max} = 259 nm; Anal. Calcd for $C_{48}H_{61}N_5O_9SSi$: C, 63.20; H, 6.74, N, 7.68. Found C, 63.19; H, 6.91; N, 7.39.

***N*1-(2,3-*O*-Isopropylidene-4-thio-5-*O*-dimethoxytritly-β-D-ribofuranosyl)-2’,3’-*O*-isopropylid**

eneadenosine (12). A solution of **11** (94 mg, 103 mmol), TBAF (1.0 M in THF, 200 mL, 0.20 mmol) and AcOH (6 μL, 1 μmol) in THF (800 mL) was stirred at room temperature for 9 h, and then evaporated. The residue was purified by column chromatography (silica gel, hexane/AcOEt = 2/1, /1, 3/2, and 1/3) to give **12** (83 mg, quant., colorless amorphous solid): ^{1}H-NMR (500 MHz, $CDCl_3$) δ 1.29 (3H, s, isopropylidene), 1.37 (3H, s, isopropylidene), 1.58 (3H, s, isopropylidene), 1.63 (3H, s, isopropylidene), 3.40 (1H, dd, *J* = 9.7, 6.9 Hz, H-5”), 3.46 (1H, dd, *J* = 9.7, 6.9 Hz, H-5”), 3.72 (1H, dd, *J* = 12.6, 10.9 Hz, H-5”), 3.77 (3H, s, OCH_3), 3.77 (3H, s, OCH_3), 3.79 (1H, m, H-4”), 3.86 (1H, d, *J* = 12.6 Hz, H-5’), 4.47 (1H, m, H-4’), 4.67 (1H, dd, *J* = 5.7 4.0 Hz, H-3”), 4.84 (1H, dd, *J* = 5.7, 2.3 Hz, H-2”), 4.99-5.03 (2H, m, H-2’, H-3’), 5.31 (1H, d, *J* = 10.9 Hz, OH), 5.75 (1H, d, *J* = 4.6 Hz, H-1’), 6.44 (1H, d, *J* = 2.3 Hz, H-1”), 6.80-7.42 (13H, m, Ar), 7.57 (1H, br s, NH), 7.63 (1H, s, H-8), 8.14 (1H, s, H-2); ^{13}C-NMR (125 MHz, $CDCl_3$) δ 25.42, 25.48, 27.60, 27.76, 54.98, 55.39, 63.28, 65.05, 67.35, 81.62, 83.90, 84.89, 86.03, 86.90, 89.28, 93.95, 112.90, 113.35, 114.39, 125.04, 127.03, 128.07, 128.26, 130.18, 130.23, 135.74, 135.92, 138.32, 139.63, 144.66, 146.01, 153.86, 158.70; UV (MeOH) λ_{max} = 258 nm; HR-MS (FAB, positive) calcd for $C_{42}H_{48}N_5O_9S$ 798.3173 [$(M+H)^+$], found 798.3193.

***N*1-(2,3-*O*-Isopropylidene-4-thio-5-*O*-dimethoxytrityl-β-D-ribofuranosyl)-5’-*O*-[bis(phenylthio)phosphoryl]-2’,3’-*O*-isopropylideneadenosine (13).** To a solution of **12** (845 mg, 1.06 mmol) in pyridine (5 mL) was added a solution of PSS (809 mg, 2.12 mmol) and TPSCl (693 mg, 1.91 mmol) in pyridine (5 mL) at -15 °C, and the mixture was stirred at the same temperature for 2.5 h. After addition of H_2O, the resulting mixture was partitioned between EtOAc and 1 M aqueous HCl, and the organic layer was washed with H_2O and brine, dried (Na_2SO_4), and evaporated. The residue was purified by column chromatography (silica gel, hexane/AcOEt = 3/1, 1/1, 1/2, and 1/3) to give **13** (812 mg, 72%, white amorphous solid): ^{1}H-NMR (500 MHz, $CDCl_3$) δ 1.25 (3H, s, isopropylidene-CH_3), 1.36 (3H, s, isopropylidene-CH_3), 1.57 (3H, s, isopropylidene-CH_3), 1.61 (3H, s, isopropylidene-CH_3), 3.40 (1H, m, H-5’), 3.46 (1H, dd, *J* = 9.2, 5.7 Hz, H-5’), 3.77 (3H, s, OCH_3), 3.77 (3H, s, OCH_3), 3.79 (1H, m , H-4”), 4.37-4.39 (2H, m, H-5”×2), 4.44 (1H, m, H-4’), 4.64 (1H, m, H-3”), 4.83 (1H, dd, *J* = 6.3, 2.3 Hz, H-2”), 4.89 (1H, dd, *J* = 6.3, 3.4 Hz, H-3’), 5.06 (1H, dd, *J* = 6.3, 2.3 Hz, H-2’), 5.97 (1H, d, *J* = 2.3 Hz, H-1’), 6.55 (1H, d, *J* = 2.3 Hz, H-1”), 6.80-7.48 (23H, m, Ar), 7.82 (1H, s, H-8), 8.09 (1H, s, H-2); ^{13}C-NMR (125 MHz, $CDCl_3$) δ 25.32, 27.14, 27.48, 55.09, 55.19, 64.99, 66.14, 66.33, 66.39, 80.89, 84.44, 84.70, 84.79, 86.58, 89.09, 90.62, 112.59, 113.12, 113.15, 123.71, 125.67, 125.71, 125.73, 125.76, 126.79, 127.85, 128.10, 129.42, 129.44, 129.63, 129.66, 129.68, 129.70, 130.03, 130.08, 135.11, 135.16, 135.27, 135.31, 135.58, 135.71, 137.27, 140.28, 144.55, 145.85, 153.96, 158.47; ^{31}P-NMR (202 MHz, $CDCl_3$) d 50.79 (s); UV (MeOH) λ_{max} = 258 nm; HR-MS (FAB, positive) calcd for $C_{54}H_{57}N_5O_{10}PS_3$ 1062.3005 [$(M+H)^+$], found 1062.2999.

***N*1-(2,3-*O*-Isopropylidene-4-thio-β-D-ribofuranosyl)-5’-*O*-[bis(phenylthio)phosphoryl]-2’,3’-*O*-isopropylideneadenosine (14).** A solution of **13** (23 mg, 22 mmol) in aqueous 60% AcOH (2 mL)

was stirred at room temperature for 4 h, and then evaporated. The residue was purified by column chromatography (silica gel, hexane/AcOEt = 1/1, 1/3, then AcOEt) to give **14** (15 mg, 90%, colorless amorphous solid): ^{1}H-NMR (500 MHz, $CDCl_3$) δ 1.34 (3H, s, isopropylidene-CH_3), 1.38 (3H, s, isopropylidene-CH_3), 1.60 (3H, s, isopropylidene-CH_3), 1.62 (3H, s, isopropylidene-CH_3), 3.76-3.83 (3H, m, H-4", H-5"×2), 4.30 (1H, ddd, *J* = 11.4, 9.7, 5.2 Hz, H-5'), 4.43 (1H, ddd, *J* = 6.3, 5.2, 2.9 Hz, H-4'), 4.47 (ddd, 1H, *J* = 12.6, 9.7, 6.3 Hz, H-5') , 4.83 (1H, d, *J* =5.2 Hz, H-3"), 5.04 (1H, dd, *J* = 6.3, 2.9 Hz, H-3'), 5.09 (1H, dd, *J* = 5.2, 1 .1 Hz, H-2") 5.28 (1H, dd, *J* = 6.3, 1.7 Hz, H-2'), 5.98 (1H, d, *J* = 1.7 Hz, H-1'), 6.07 (1H, d, *J* = 1.1 Hz , H-1"), 7.27-7.54 (10H, m, Ar), 7.63 (1H, s, H-8), 8.55 (1H, s, H-2); ^{13}C-NMR (125 MHz, $CDCl_3$) d 24.88, 25.08, 26.87, 27.28, 57.09, 64.26, 66.26, 66.32, 73.96, 81.93, 84.52, 85.65, 85.73, 85.93, 89.68, 91.39, 110.99, 114.10, 123.84, 125.28, 125.34, 125.46, 125.52, 129.27, 129.29, 129.38, 129.40, 129.58, 129.60, 129.73, 129.75, 135.04, 135.09, 135.37, 135.40, 138.01, 140.22, 147.09, 154.09; ^{31}P-NMR (202 MHz, $CDCl_3$) d 52.23 (s); UV (MeOH) λ_{max} = 251, 258 nm; HR-MS (FAB, positive) calcd for $C_{33}H_{39}N_5O_8PS_3$ 760.1698 [$(M+H)^+$], found 760.1710.

***N*1-(2,3-*O*-Isopropylidene-4-thio-5-*O*-phospholyl-β-D-ribofuranosyl)-5'-*O*-(phenylthiophosphoryl)-2',3'-*O*-isopropylideneadenosine (16).** A solution of $MeOPOCl_2$ (30 mL, 0.30 mmol) in pyridine (1 mL) was stirred at -15 °C for 15 min. To the solution was added a solution of **14** (75 mg, 0.10 mol) in pyridine (1 mL) , and the mixture was stirred at the same temperature for 2h. To the resulting solution was added triethylammonium acetate (TEAA) buffer (0.5 M, pH7.0, 3 mL) then H_3PO_2 (101 μL, 2.0 mmol) and Et_3N (140 μL, 1.0 mmol), and the mixture was stirred at room temperature for 13 h, and then evaporated. The residue was partitioned between EtOAc and H_2O, and the aqueous layer was evaporated. The residue was purified by column chromatography (ODS, 1.2 x 16 cm, 0-37% CH_3CN /0.1M TEAA buffer (0.1 M, pH 7.0, 400 mL), linear gradient). The excess TEAA included in the residue was removed by column chromatography (ODS, 1.2 x 16 cm, CH_3CN/H_2O = 1/1). The product was lyophilized to give **16** (37 mg, 512 OD_{260} unit, 46%) as a triethylammonium salt: ^{1}H-NMR (500 MHz, D_2O) δ 1.26 (9H, t, *J* = 7.4 Hz $(CH_3CH_2)_3N$), 1.40 (3H, s, isopropylidene-CH_3), 1.43 (3H, s, isopropylidene-CH_3), 1.63 (3H, s, isopropylidene-CH_3), 1.69 (3H, s, isopropylidene-CH_3), 3.18 (6H, q, *J* = 7.4 Hz, $(CH_3CH_2)_3N$), 4.10-4.13 (4H, m, H-4", H-5'×2, H-5"), 4.70 (1H, m, H-4"), 4.20 (1H, m, H-5'), 4.22 (1H, m, H-5"), 4.32 (1H, m, H-5"), 4.71 (1H, m, H-4'), 4.94 (1H, d, *J* = 5.2 Hz, H-3') 5.12-5.15 (2H, m, H-2", H-3"), 5.42 (1H, dd, *J* = 6.3, 2.9 Hz, H-3'), 5.39 (1H, d, *J* = 5.2 Hz, H-2'), 5.95 (1H, s, H-1"), 6.37 (1H, s, H-1'), 7.19-7.33 (5H, m, Ar), 8.41 (1H, s, 8-H), 9.24 (1H, s, 2-H); ^{13}C-NMR (125 MHz, D_2O) δ 8.30, 24.32, 24.35, 25.95, 26.41, 46.71, 54.48, 54.55, 65.90, 65.95, 66.59, 66.62, 75.84, 81.39, 84.11, 86.19, 86.27, 86.52, 89.30, 90.88, 113.51, 114.72, 119.08, 127.91, 129.08, 129.47, 129.51, 132.75, 132.79, 143.02, 145.48, 146.84, 150.53; ^{31}P-NMR (202 MHz, D_2O) d 17.54 (s), 0.80 (s); UV (D_2O) λ_{max} = 258 nm; HR-MS (FAB, negative) calcd for $C_{27}H_{34}N_5O_{12}P_2S_2$ 746.1126 [$(M-H)^-$], found 746.1106.

Cyclic ADP-4-thio-ribose 2',3'-, 2",3"-bisacetonide (17). To a mixture of $AgNO_3$ (36 mg, 0.21

mmol), Et_3N (29 ml, 0.21 mmol), and MS 3A (powder, 1.0 g) in pyridine (8 mL), a solution of **16** (9 mg, 50 OD_{260} unit, 4 mmol) in pyridine (8 mL) was added slowly over 15 h, using a syringe-pump, at room temperature under shading. To the mixture was added TEAA buffer (2.0 M, pH 7.0, 2 mL), and the resulting mixture was filtered with Celite and evaporated. The residue was partitioned between EtOAc and H_2O, and the aqueous layer was evaporated, and the residue was purified by column chromatography (ODS, 1.2 x 16 cm, 0-35% CH_3CN /0.1M TEAA buffer (0.1 M, pH 7.0, 400 mL), linear gradient). The excess TEAA included in the residue was removed by column chromatography (ODS, 1.2 x 16 cm, CH_3CN/H_2O = 1/1). The product was lyophilized to give **17** (6 mg, 36 OD_{260} unit, 72%) as a triethylammonium salt: ^{1}H-NMR (500 MHz, D_2O) δ 1.24 (9H, t, *J* = 7.4 Hz ($CH_3CH_2)_3N$), 1.41 (3H, s, isopropylidene-CH_3), 1.43 (3H, s, isopropylidene-CH_3), 1.61 (3H, s, isopropylidene-CH_3), 1.65 (3H, s, isopropylidene-CH_3), 3.17 (6H, q, *J* = 7.4 Hz, $(CH_3CH_2)_3N$), 3.91 (1H, m, H-5'), 4.13 (1H, m, H-4"), 4.20 (1H, m, H-5'), 4.22 (1H, m, H-5"), 4.32 (1H, m, H-5"), 4.58 (1H, m, H-4'), 5.07 (1H, d, *J* = 4.8 Hz, H-3") 5.13 (1H, d, *J* = 4.8 Hz, H-2"), 5.42 (1H, dd, *J* = 6.3, 2.9 Hz, H-3'), 5.89 (1H, dd, *J* = 6.3, 1.7 Hz, H-2'), 5.99 (1H, s, H-1"), 6.38 (1H, d, *J* = 1.7 Hz, H-1'), 8.38 (1H, s, 8-H), 9.59 (1H, s, 2-H); ^{13}C-NMR (125 MHz, D_2O) δ 8.29, 24.39, 24.42, 26.07, 26.26, 46.73, 55.40, 55.49, 64.80, 68.03, 64.83, 68.00, 77.65, 81.36, 83.25, 86.65, 86.73, 87.34, 90.82, 91.45, 113.11, 114.59, 119.58, 144.91, 145.55, 147.20, 150.67; ^{31}P-NMR (202 MHz, D_2O) d -10.62 (d, *J* = 15.5 Hz), d -11.29 (d, *J* = 15.5 Hz); UV (D_2O) λ_{max} = 258 nm; HR-MS (FAB, negative) calcd for $C_{21}H_{28}N_5O_{12}P_2S$ 636.0936 [$(M-H)^-$], found 636.0947.

Cyclic ADP-4-thio-ribose (3). A solution of **17** (280 OD_{260} unit, 25 mmol) in aqueous 60% HCO_2H (1 mL) was stirred at room temperature for 40 h and then evaporated. After co-evaporation with H_2O, the residue was purified by column chromatography (ODS, 1.2 x 16 cm, TEAA buffer (5 mM, pH 7.0)). The excess TEAA included in the residue was removed by column chromatography (Sephadex LH 20, 2.5 x 20 cm, H_2O) to give **3** as a triethylammonium salt, which was converted into a free acid form by passing through columns of Daiaion PK212L (H^+ form, 0.7 cm × 5 cm, H_2O). The free acid was passed throuh Chelex 100 column (K^+ form, 0.7 cm × 6 cm, H_2O). The eluent was evapolated and lyophilized to give **3** (138 OD_{260} unit, 49%) as a potassium salt. **3** (potassium salt): ^{1}H-NMR (500 MHz, D_2O) δ 3.59 (1H, dd, *J* = 8.2, 3.2 Hz), 4.08 (1H, m), 4.19 (1H, m), 4.24-4.36 (4H, m), 4.52 (1H, ddd *J* = 10.4, 8.2, 2.7 Hz), 4.60 (1H, dd, *J* = 5.4, 2.3 Hz), 5.18 (1H, dd, *J* = 5.9, 5.4 Hz), 5.87 (2H, m), 7.97 (1H, s), 9.25 (1H, s); ^{13}C-NMR (125 MHz, D_2O) δ 50.8, 50.9, 63.1, 65.0, 65.0, 70.1, 70.6, 72.6, 73.0, 78.3, 84.7, 84.8, 90.5, 120.0, 145.3, 145.9, 146.9, 150.8; ^{31}P-NMR (162 MHz, D_2O) δ -9.15 (d, *J* = 12.2 Hz), δ -10.20 (d, *J* = 12.2 Hz); UV (D_2O) λ_{max} = 258 nm; HR-MS (FAB, negative) calcd for $C_{15}H_{20}N_5O_{12}P_2S$ 556.0304 [$(M-H)^-$], found 556.2994. **3** (free acid): ^{1}H-NMR (500 MHz, D_2O) δ 3.72 (1H, ddd, *J* = 5.2, 3.4, 1.7 Hz), 4.09 (1H, ddd, *J* = 10.9, 6.9, 2.9 Hz), 4.25 (1H, ddd, *J* = 11.5, 5.2, 2.3 Hz), 4.35-4.39 (3H, m), 4.51 (1H, ddd, *J* = 10.9, 7.4, 4.0 Hz), 4.53 (1H, dd, *J* = 5.9, 2.9 Hz), 4.71 (1H, dd, *J* = 5.2, 2.3 Hz), 5.22 (1H, dd, *J* = 6.3, 5.2 Hz), 5.97 (1H, d, *J* = 2.9 Hz), 6.04 (1H,

d, J = 6.3 Hz), 8.38 (1H, s), 9.90 (1H, s).

Computational Calculations. For structure determination, molecular calculations were carried out by AMBER11 (Case, D. A.; Darden, T.A.; Cheatham, T. E. III; Simmerling, C. L.; Wang, J.; Duke, R. E.; Luo, R.: Walker, R. C. ; Zhang, W. ; Merz, K. M.; Roberts, B. ; Wang, B. ; Hayik, S. ; Roitberg, A. ; Seabra, G.; Kolossvary, I.; Wong, K.F.; Paesani, F.; Vanicek, J.; Wu, X.; Brozell, S. R.; Steinbrecher, T.; Gohlke, H.; Cai, Q.; Ye, X.; Wang, J.; Hsieh, M.-J.; Cui, G.; Roe, D. R.; Mathews, D. H.; Seetin, M. G.; Sagui, C.; Babin, V.; Luchko, T.; Gusarov, S.; Kovalenko, A.; Kollman, P.A. AMBER 11, University of California, San Francisco, 2010). cADPR (**1**), cADPcR (**2**) and cADPtR (**3**) were modeled by the General AMBER Force Field (GAFF: Wang, J.; Wolf, R. M.; Caldwell, J. W.; Kollman, P. A.; Case, D. A. *J. Comput. Chem.* **2004**, *25*, 1157-1174). AM1-BCC charge (Jakalian, A.; Jack, D. B.; Bayly, C. I. *J. Comput. Chem.* **2002**, *23*, 1623-1641) was used for cADPR and its analogs assigned by Antechamber module (Wang, J.; Wang, W.; Kollman, P. A.; Case, D. A. *J Mol Graph Model* **2006**, *25*, 247-260) of AMBER11. In the calculations, interatomic distances restricts were determined by the integrated volumes of the NOESY cross-peaks. As cross peaks of cADPR and cADPcR, previously published data (Kudoh, T.; Fukuoka, M.; Ichikawa, S.; Murayama, T.; Ogawa, Y.; Hashii, M.; Higashida, H.; Kunerth, S.; Weber, K.; Guse, A. H.; Potter, B. V.; Matsuda, A.; Shuto, S. *J. Am. Chem. Soc.* **2005**, *127*, 8846-8855) were used[5]. For generating conformations, we carried out 400 ps simulated annealing molecular dynamics simulations: temperature was decreased from 1000 to 300 K. These simulations were repeated 100 times with different initial velocity for each compound. In calculated conformations, we chose and analyzed the lowest energy conformation.

a) cADPR (**1**) b) cADPcR (**2**) c) 3cADPtR (**3**)

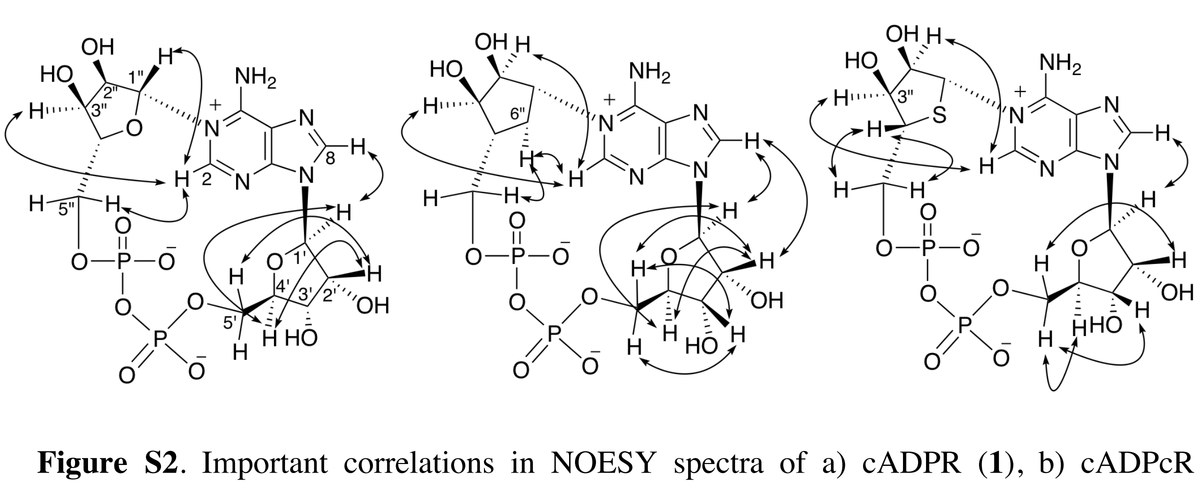

Figure S2. Important correlations in NOESY spectra of a) cADPR (**1**), b) cADPcR (**2**), c) cADPtR (**3**), used for the calculations.

Stability in Rat Brain Microsomes. Rat brain microsomes were prepared by a procedure according to the previous method (Murayama T.; Ogawa Y.; *J. Biol. Chem.* **1996**, *271*, 5079-5084.). cADPR or cADPtR (1.6 OD_{260}unit) was preincubated in 20 mM MOPS buffer (pH 7.1, 160 μL) at

37 °C for 5 min. This was added to the solution of the microsome fraction of rat brain extract (14.9 mg/ mL, 140 μL), and the mixture was incubated at 37 °C. The reaction mixture was sampled (25 μL) at every 30 min afterwards and diluted with water (175 μL), which was frozen in liquid nitrogen to stop the reaction. After the samples were a centrifuged at 12000 rpm at 4 °C for 15 min, the supernatants were filtered using centrifugal filter at 12000 rpm at 4 °C for 15 min, and the resulting filtrates (70 μL) were analyzed by ion exchange HPLC (TSK-GEL DEAE-2SW, 4.6 x 250 mm; 5-35% 1M HCO_2NH_4/20% MeCN, 20 min; 260 nm). The results are shown in Figure S2.

Biological Evaluations with Sea Urchin Egg Homogenate or T-cells. These bioassays were carried out as reported previously (Kudoh, T.; Fukuoka, M.; Ichikawa, S.; Murayama, T.; Ogawa, Y.; Hashii, M.; Higashida, H.; Kunerth, S.; Weber, K.; Guse, A. H.; Potter, B. V.; Matsuda, A.; Shuto, S. *J. Am. Chem. Soc.* **2005**, *127*, 8846-8855).

Biological Evaluations with Neuronal Cells Culture. NG108-15 neuroblastoma x glioma hybrid cells were cultured as reported previously (Higashida H, Hashii M, Fukuda K, Caulfield MP, Numa S, Brown D. A. *Proc. Biol. Sci. 242*, **1990**, 68-74). Oregon Geen-loaded NG108-15 cells were incubated for 2 min in the following calcium-free medium (140 mM K-glutamate, 20 mM PIPES, 5 mM EGTA, 2 mM Mg-ATP, 10 mM glucose, 1 mM magnesium chloride, 0.01% bovine serum albumin, pH 6.8) at 37°C, and subsequently permeabilized with 250 nM digitonin in the calcium-free medium. cADPR (1-100 μM), cADPcR (1-100 μM), or cADPcR (1-100 μM) were applied together with the digitonin-containing permeabilization buffer to be allowed free passage of these nucleotides into the cytoplasm. Concentrations of $[Ca^{2+}]_i$ were determined microspectrofluorometrically using fura-2 in differentiated NG108-15 cells cultured on polylysine-coated glass coverslips. The cells were loaded with fura-2 using 5 μM Oregon Green 488 1,2-bis(2-aminophenoxy)ethane-N,N,N,N-tetraacetate acetoxymethylester (BAPTA-1 AM). Fluorescence was measured at 37°C with excitation wavelengths of 485 nm and emission wavelengths of 538 nm using an Argus 50 Ca^{2+} microspectrofluorometric system (Hamamatsu Photonics, Hamamatsu, Japan) and images were collected every 10 s for up to 5 min. The changes in fluorescence intensity of each cell were expanded into an X-t plane, and data were performed in fluorescence intensity at each time (*x)* divided by resting intensity at time 0, i.e., F_x/F_0. (Amina, S.; Hashii, M.; Ma, W. J.; Yokoyama, S.; Lopatina, O.; Liu, H. X.; Islam, M. S.; Higashida, H. *J. Neuroendocrinol.* **2010**, *22*, 460-466.) Data are expressed as mean ± s.e.m. Statistical analysis was performed using a Student's t test. The criterion for significance in all cases was $p<0.05$.

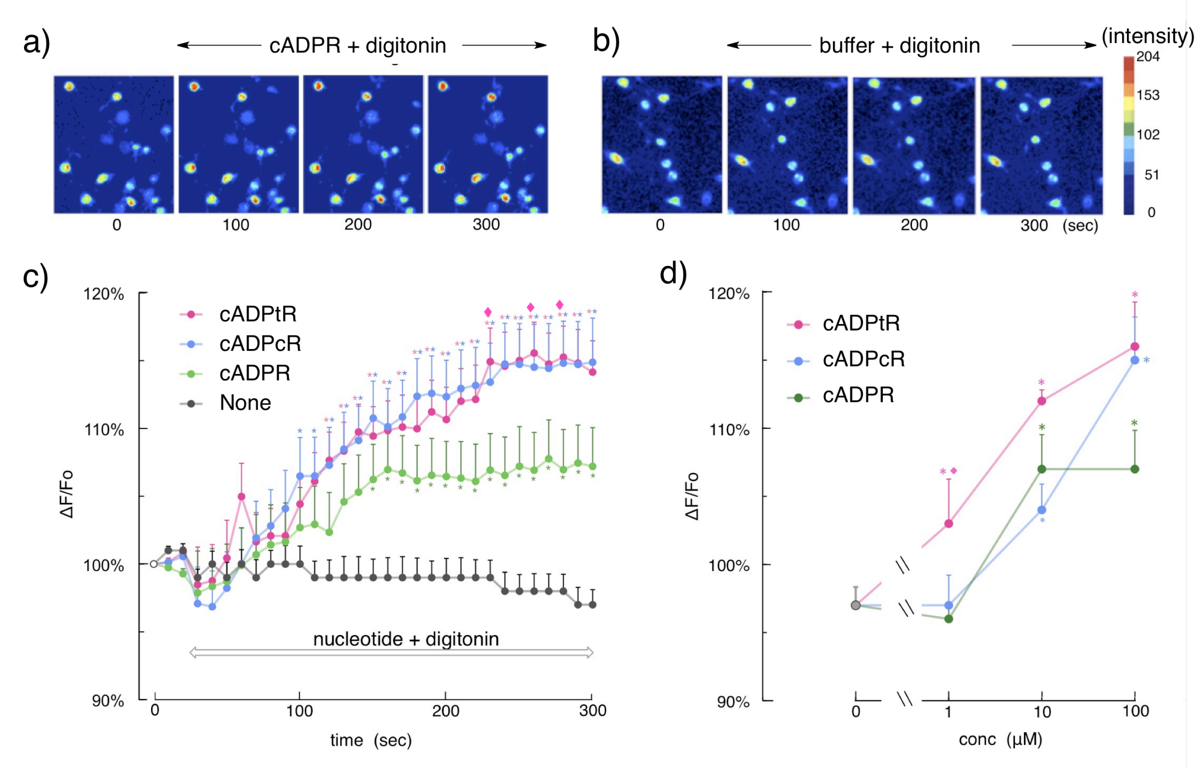

Figure S3. Effects of cADPtR on $[Ca^{2+}]_i$ increases in permeabilized NG108-15 cells. a), b) Cells were permeabilized by the addition of 250 nM digitonin to the bath solution. cADPtR (final concentration, 100 μM) (a) or an equal amount of buffer (b) was added together with digitonin as indicated by the arrows. Representative fields are displayed for each condition. Changes in $[Ca^{2+}]_i$ are shown as pseudocolor images, and colors reflecting fluorescence intensities are indicated to the right together with arbitrary units. c) Time-course of $[Ca^{2+}]_i$ changes in Oregon Green-loaded NG 108-15 cells. At about 25 s after the beginning of each trace, cell membranes were permeabilized with buffers containing 250 nM digitonin with 100 μM cADPtR (magenta), cADPcR (blue), cADPR (green), or without nucleotide as control (black). Symbols indicate changes of $[Ca^{2+}]_i$ levels for 5 min, represented by the fluorescence intensity at each time (x) divided by resting intensity at time 0 (i.e. F_x/F_0). For calculations, cells with mean fluorescence intensity of 90 to 130 at time 0 were selected. *, Values in cells treated with cADPtR or cADPcR were significantly higher than those in control cells at p <0.05. ♦, Values in cells treated with cADPtR were significantly higher than those in cells treated with cADPR at p <0.05. d) The graph shows concentration-dependent activity of cADPtR (magenta), cADPcR (blue) and cADPR (green) in NG108-15 cells. Symbols indicate changes of $[Ca^{2+}]_i$ levels at 280 s after membrane permeabilization, represented by fluorescence intensity at each time divided by the resting state at time 0 (i.e. F_x/F_0). Each symbol is mean ± SEM of 5 to 10 experiments. *, values significantly different from a drug-free control value (a gray circle) ($P < 0.05$). ♦, Values in cells treated with cADPtR were significantly higher than those in cells with cADPR at p <0.05.

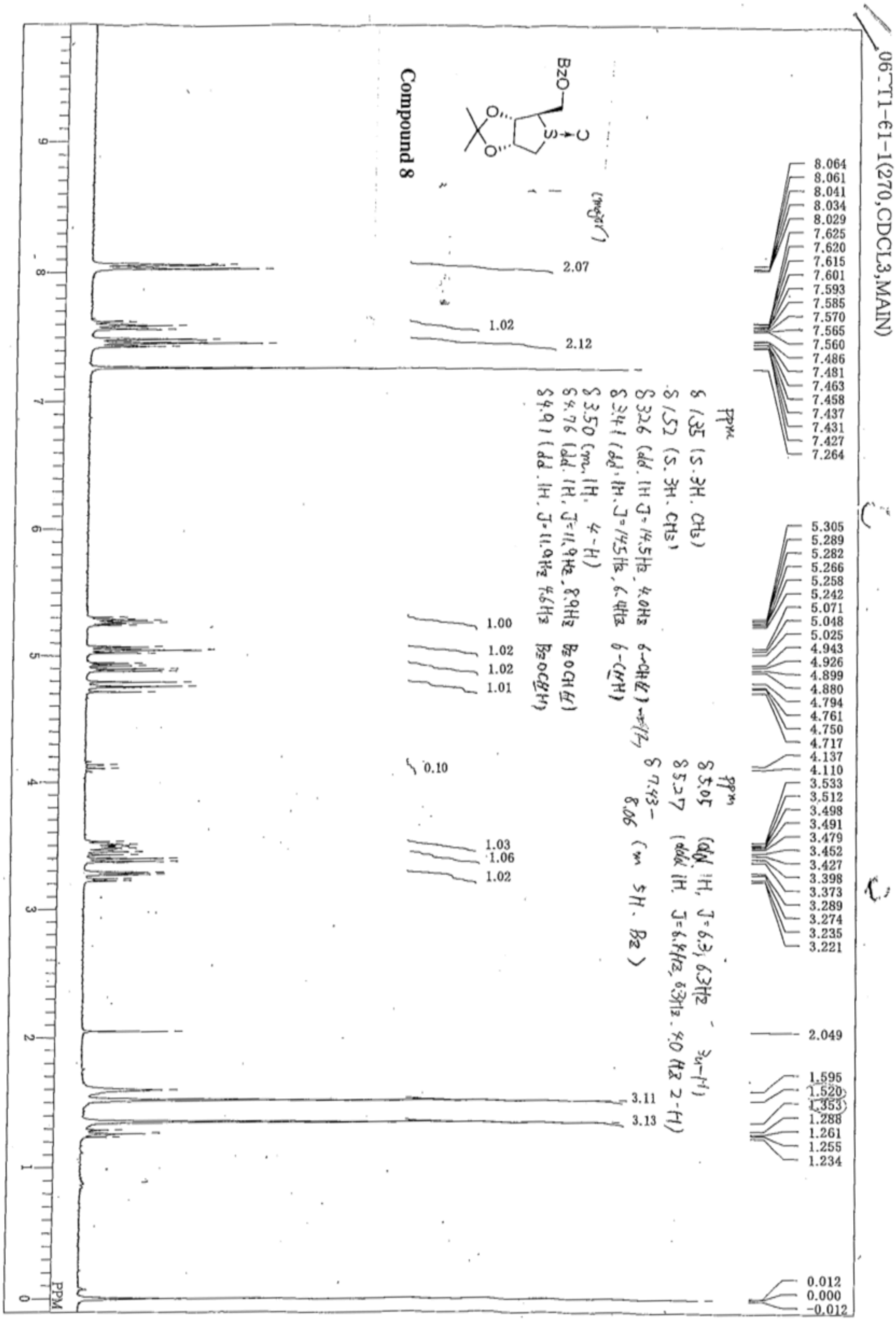
06-TI-61-1(270,CDCL3,MAIN)
Compound 8
BzO
PPM

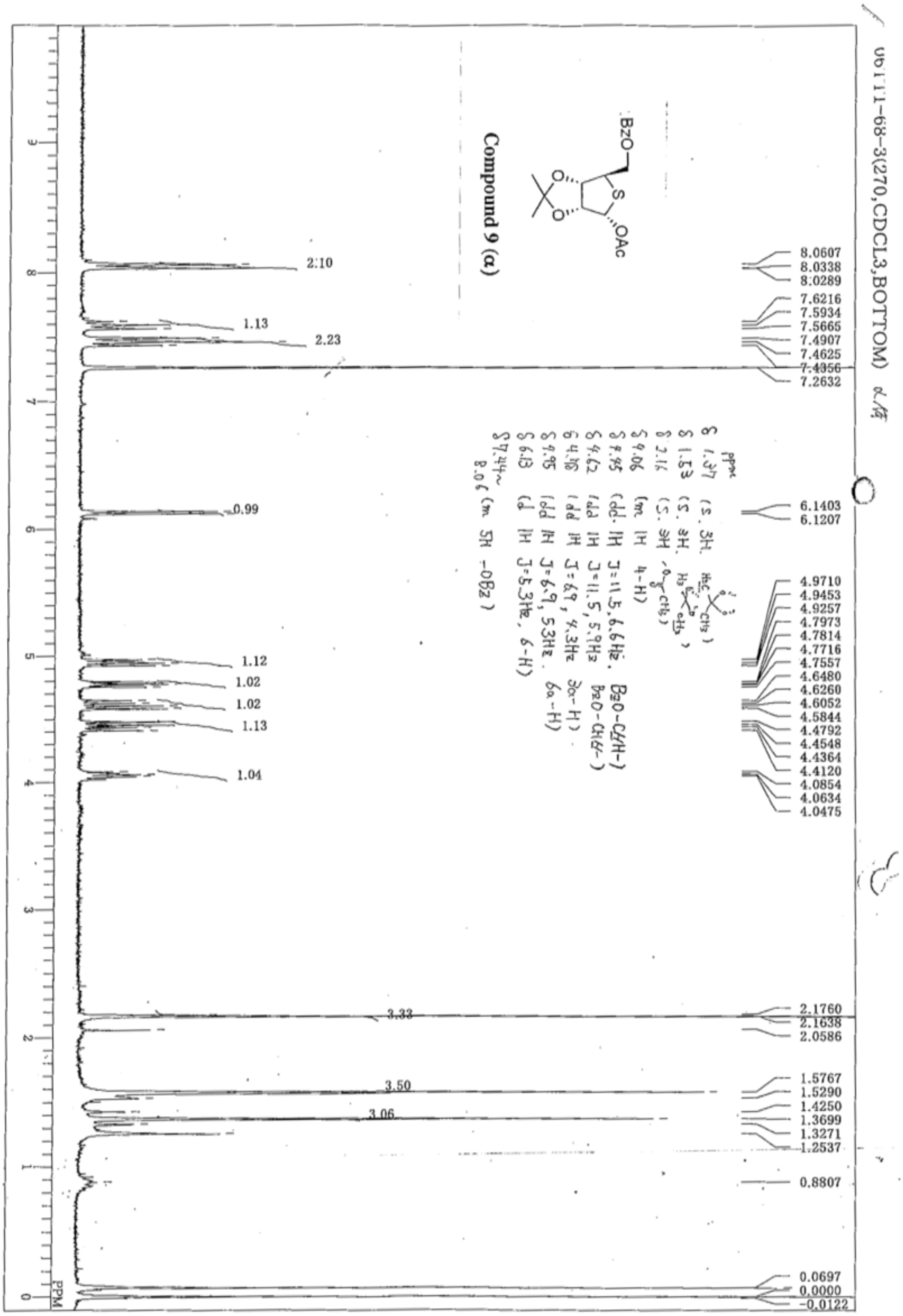
u6111-68-3(270,CDCL3,BOTTOM)
Compound 9 (α)
BzO
S
OAc
O
O
8.0607
8.0338
8.0289
7.6216
7.5934
7.5665
7.4907
7.4625
7.4356
7.2632
6.1403
6.1207
4.9710
4.9453
4.9257
4.7973
4.7814
4.7716
4.7557
4.6480
4.6260
4.6052
4.5844
4.4792
4.4548
4.4364
4.4120
4.0854
4.0634
4.0475
2.1760
2.1638
2.0586
1.5767
1.5290
1.4250
1.3699
1.3271
1.2537
0.8807
0.0697
0.0000
-0.0122
2.10
1.13
2.23
0.99
1.12
1.02
1.02
1.13
1.04
3.33
3.50
3.06
PPM

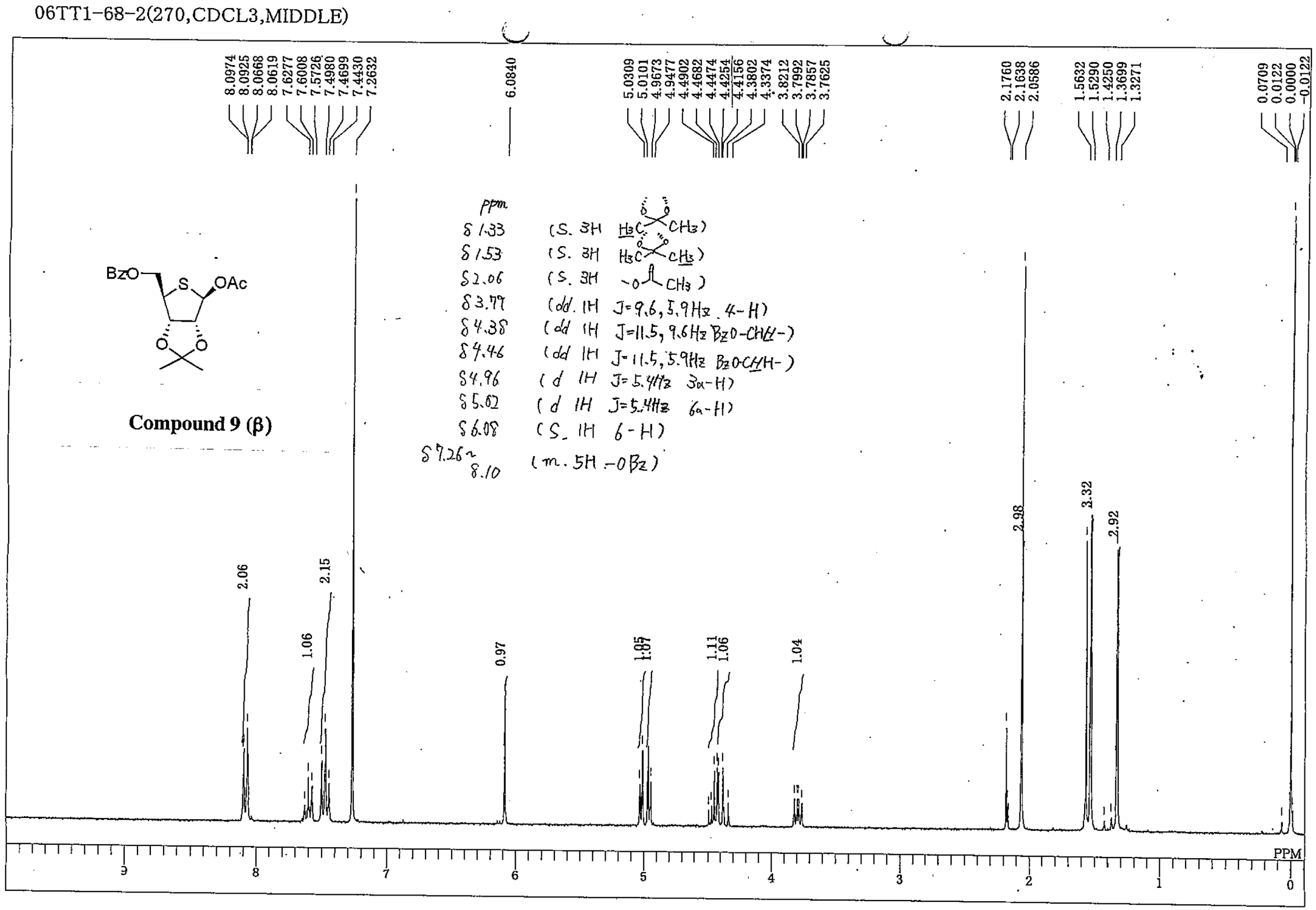
06TT1-68-2(270,CDCL3,MIDDLE)
BzO
S
OAc
O
O
8.0974
8.0925
8.0668
8.0619
7.6277
7.6008
7.5726
7.4980
7.4699
7.4430
7.2632
6.0840
5.0309
5.0101
4.9673
4.9477
4.4902
4.4682
4.4474
4.4254
4.4156
4.3802
4.3374
3.8212
3.7992
3.7857
3.7625
2.1760
2.1638
2.0586
1.5632
1.5290
1.4250
1.3699
1.3271
0.0709
0.0122
0.0000
-0.0122
ppm
δ 1.33 (S. 3H H3C CH3)
δ 1.53 (S. 3H H3C CH3)
δ 2.06 (S. 3H -O CH3)
δ 3.77 (dd. 1H J=9.6, 5.9Hz 4-H)
δ 4.38 (dd 1H J=11.5, 9.6Hz BzO-CHH-)
δ 4.46 (dd 1H J=11.5, 5.9Hz BzO-CHH-)
δ 4.96 (d 1H J=5.4Hz 3a-H)
δ 5.02 (d 1H J=5.4Hz 6a-H)
δ 6.08 (S 1H 6-H)
δ 7.26~8.10 (m. 5H -OBz)
2.06
1.06
2.15
0.97
1.05
1.07
1.11
1.06
1.04
2.98
3.32
2.92
PPM
9
8
7
6
5
4
3
2
1
0

Compound 9 (β)

06TT1-85-1(500,CDCl3)

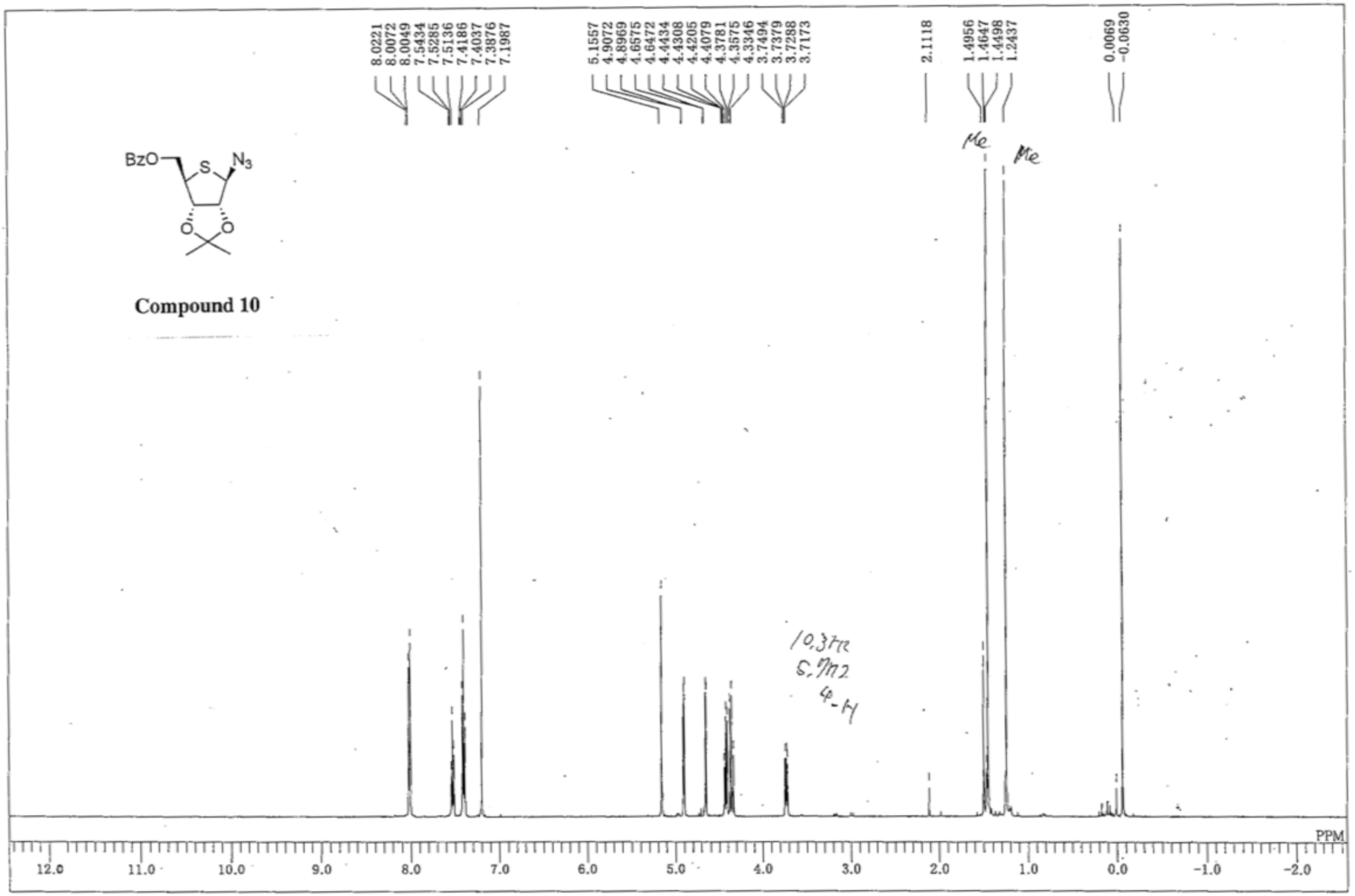

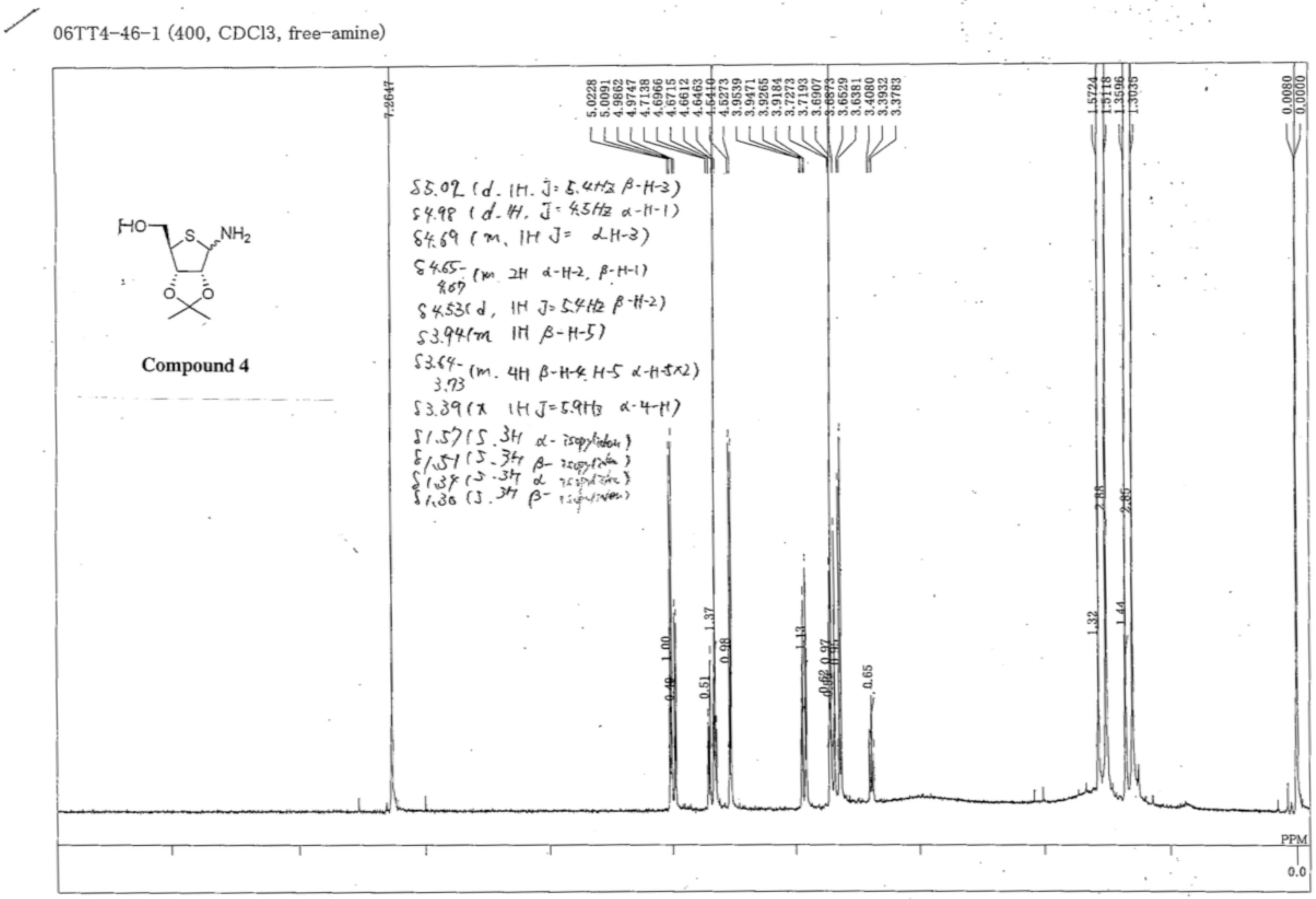
06TT4-46-1 (400, CDCl3, free-amine)
HO
S
NH2
O
O
Compound 4
7.2647
5.0228
5.0091
4.9862
4.9747
4.7138
4.6966
4.6715
4.6612
4.6463
4.5410
4.5273
3.9539
3.9471
3.9265
3.9184
3.7273
3.7193
3.6907
3.6873
3.6529
3.6381
3.4080
3.3932
3.3783
1.5724
1.5118
1.3596
1.3035
0.0080
0.0000
1.00
0.51
1.37
0.98
1.13
0.97
0.65
1.32
2.88
1.44
2.85
PPM
0.0

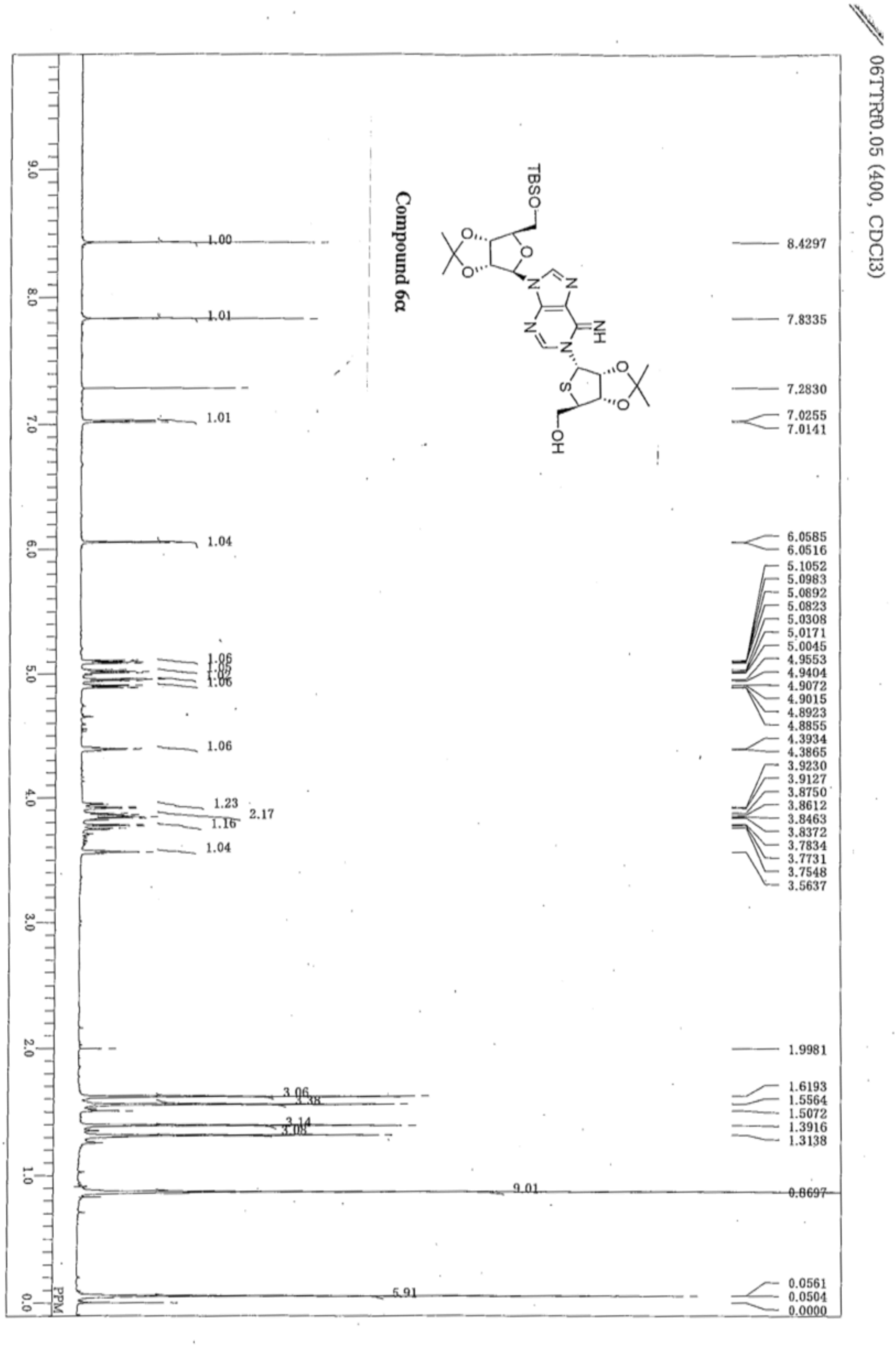
06TTRf0.05 (400, CDCl3)
Compound 6α
TBSO
NH
OH
S
8.4297
7.8335
7.2830
7.0255
7.0141
6.0585
6.0516
5.1052
5.0983
5.0892
5.0823
5.0308
5.0171
5.0045
4.9553
4.9404
4.9072
4.9015
4.8923
4.8855
4.3934
4.3865
3.9230
3.9127
3.8750
3.8612
3.8463
3.8372
3.7834
3.7731
3.7548
3.5637
1.9981
1.6193
1.5564
1.5072
1.3916
1.3138
0.8697
0.0561
0.0504
0.0000
1.00
1.01
1.01
1.04
1.06
1.06
1.23
2.17
1.16
1.04
3.06
3.14
9.01
5.91
PPM

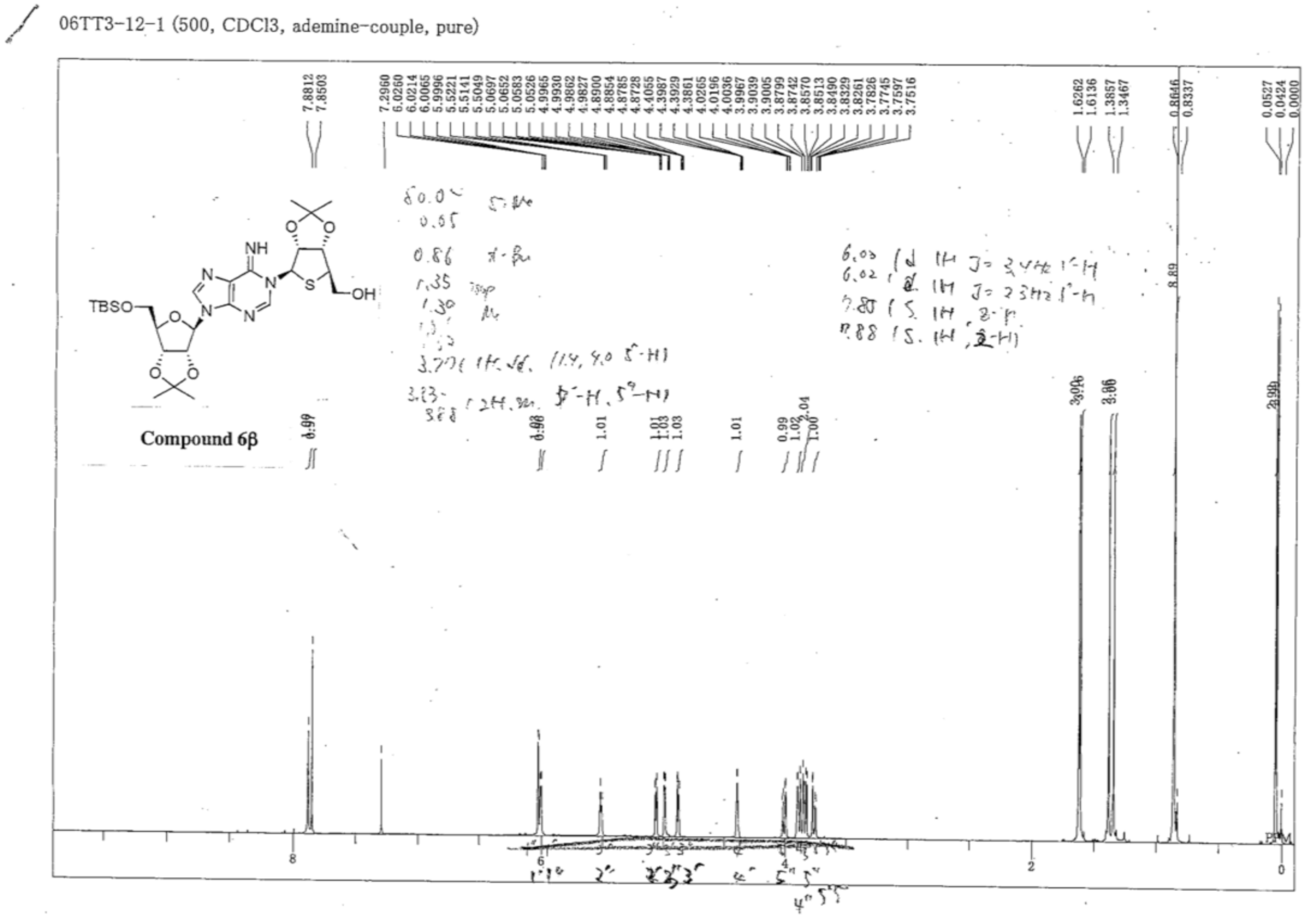
06TT3-12-1 (500, CDCl3, ademine-couple, pure)
7.8812
7.8503
7.2960
6.0260
6.0214
6.0065
5.9996
5.5221
5.5141
5.5049
5.0697
5.0652
5.0583
5.0526
4.9965
4.9930
4.9862
4.9827
4.8900
4.8854
4.8785
4.8728
4.4055
4.3987
4.3929
4.3861
4.0265
4.0196
4.0036
3.9967
3.9039
3.9005
3.8799
3.8742
3.8570
3.8513
3.8490
3.8329
3.8261
3.7826
3.7745
3.7597
3.7516
1.6262
1.6136
1.3857
1.3467
0.8646
0.8337
0.0527
0.0424
0.0000
TBSO
NH
OH
S
Compound 6β
8.89
8
6
2
0

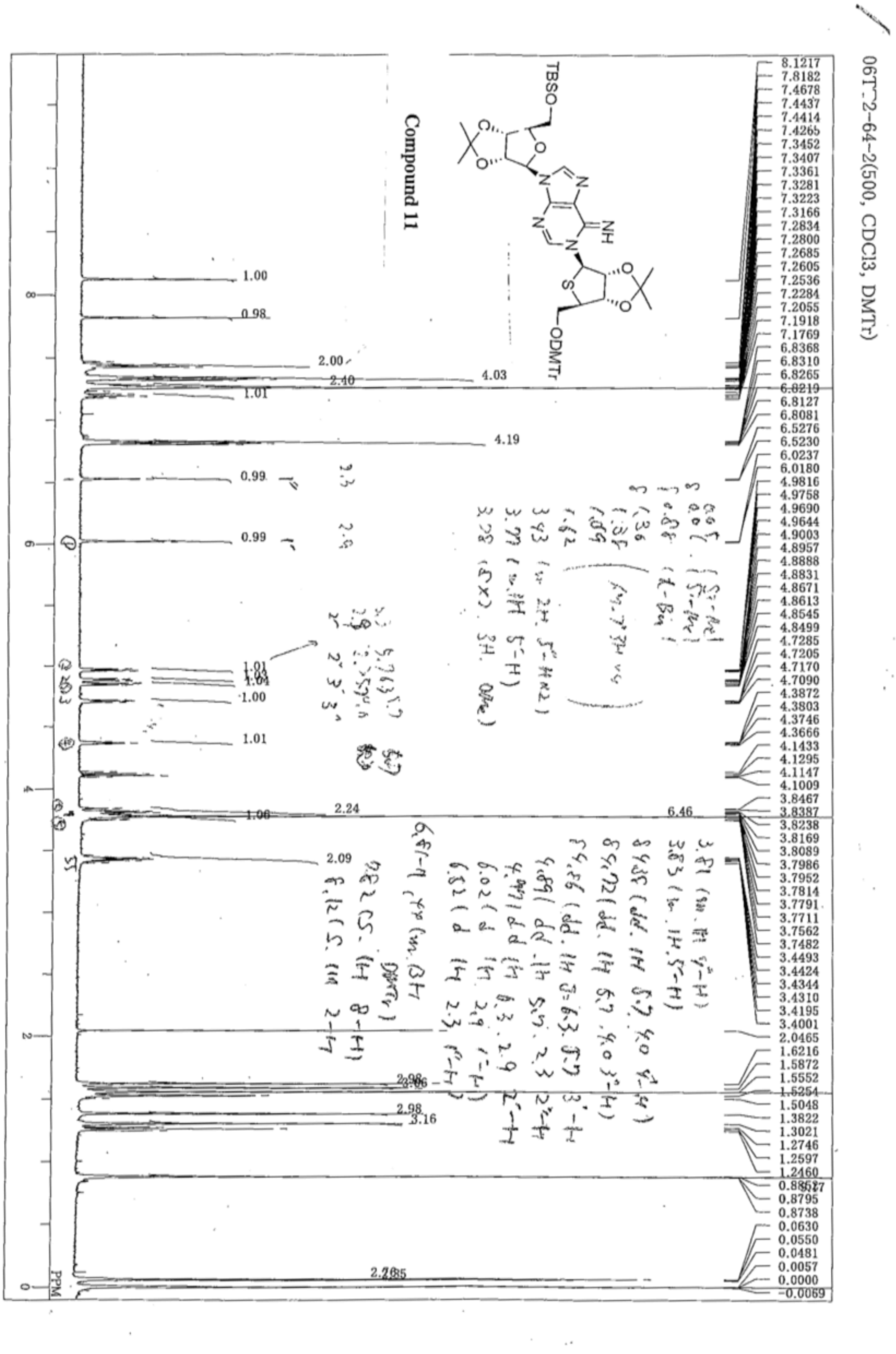
06T˜2-64-2(500, CDCl3, DMTr)
Compound 11
TBSO
ODMTr
NH
PPM

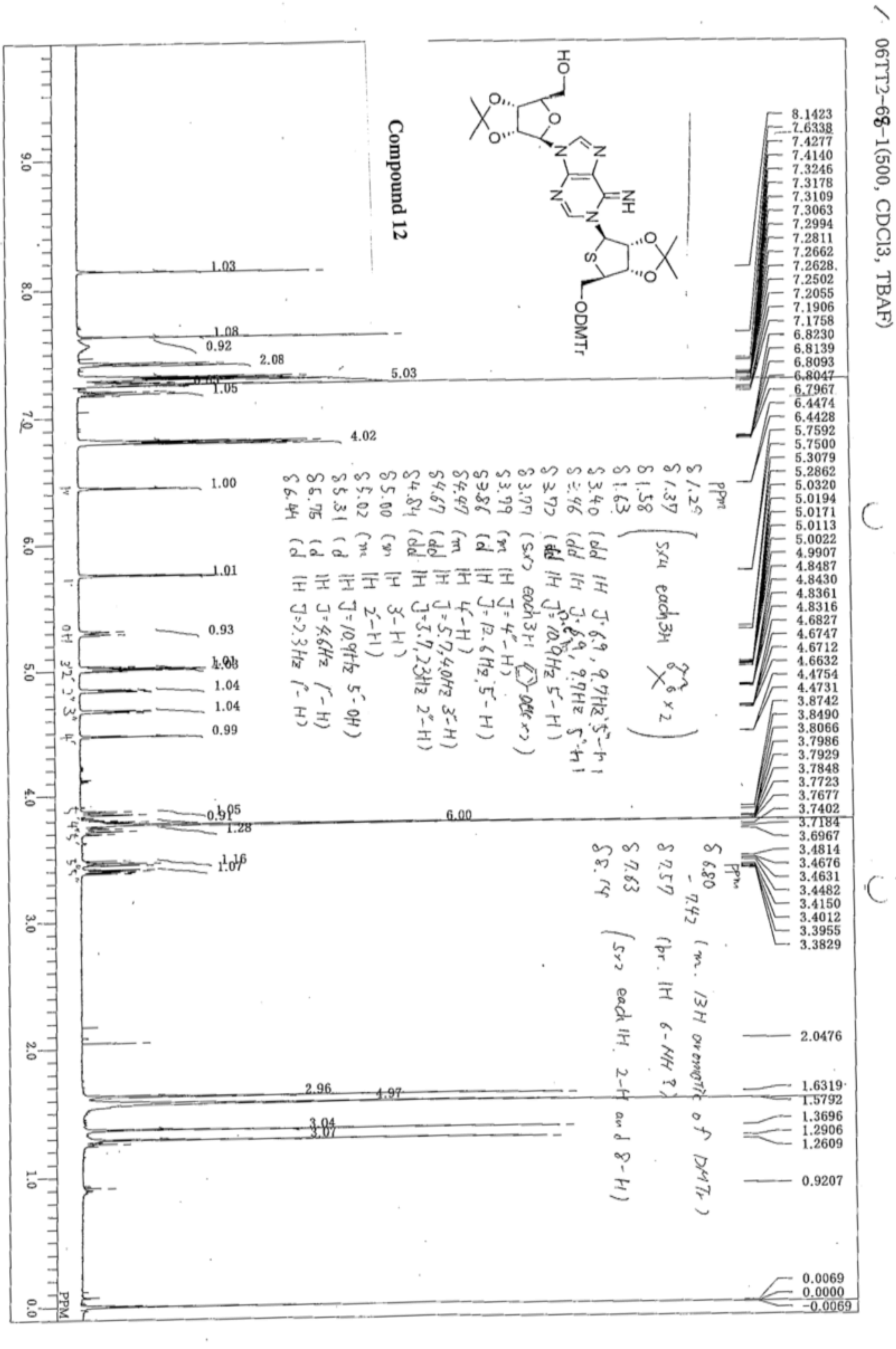
06TT2-68-1(500, CDCl3, TBAF)
Compound 12

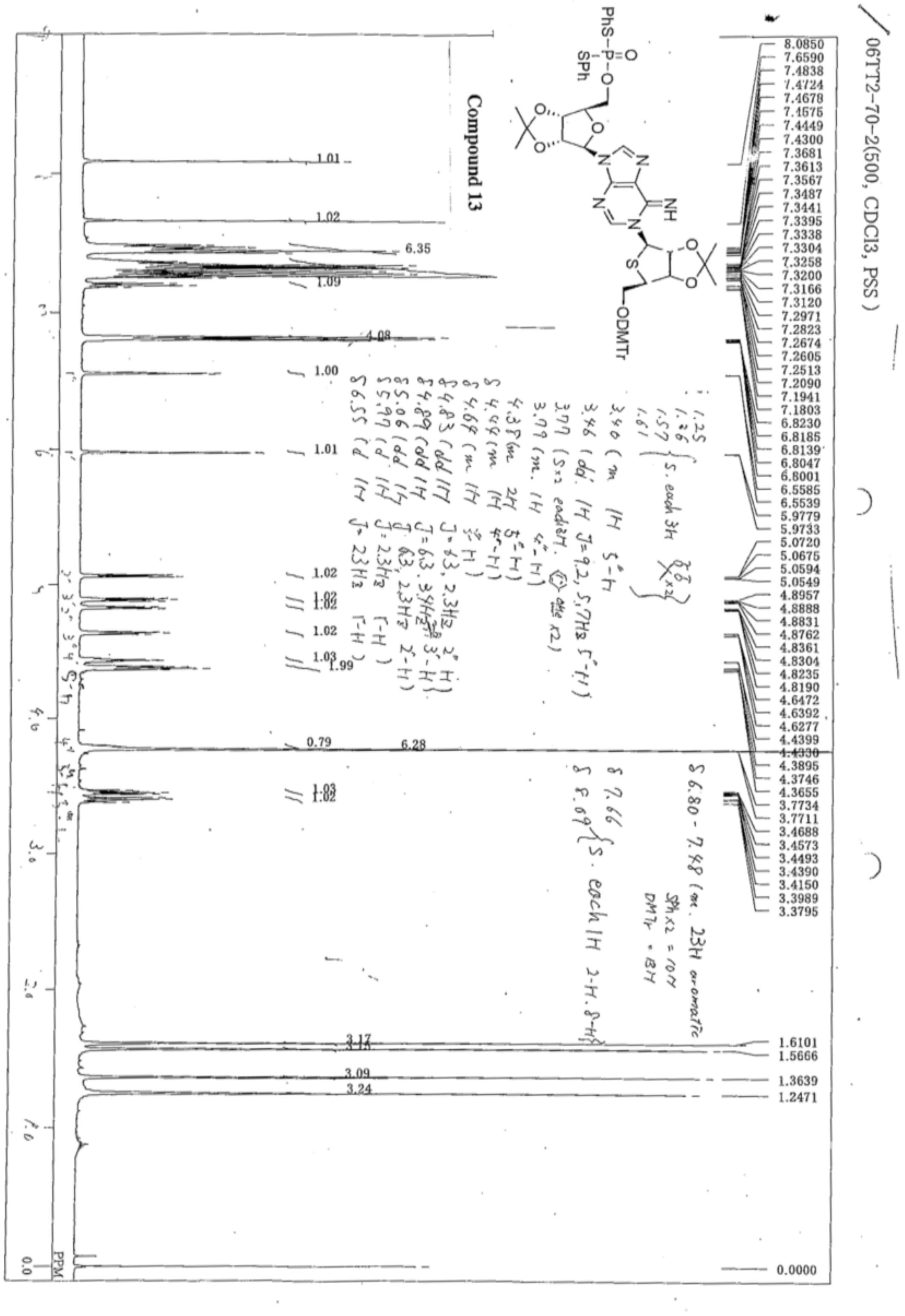
06TT2-70-2(500, CDCl3, PSS)
Compound 13
8.0850
7.6590
7.4838
7.4724
7.4678
7.4575
7.4449
7.4300
7.3681
7.3613
7.3567
7.3487
7.3441
7.3395
7.3338
7.3304
7.3258
7.3200
7.3166
7.3120
7.2971
7.2823
7.2674
7.2605
7.2513
7.2090
7.1941
7.1803
6.8230
6.8185
6.8139
6.8047
6.8001
6.5585
6.5539
5.9779
5.9733
5.0720
5.0675
5.0594
5.0549
4.8957
4.8888
4.8831
4.8762
4.8361
4.8304
4.8235
4.8190
4.6472
4.6392
4.6277
4.4399
4.4330
4.3895
4.3746
4.3655
3.7734
3.7711
3.4688
3.4573
3.4493
3.4390
3.4150
3.3989
3.3795
1.6101
1.5666
1.3639
1.2471
0.0000
PPM

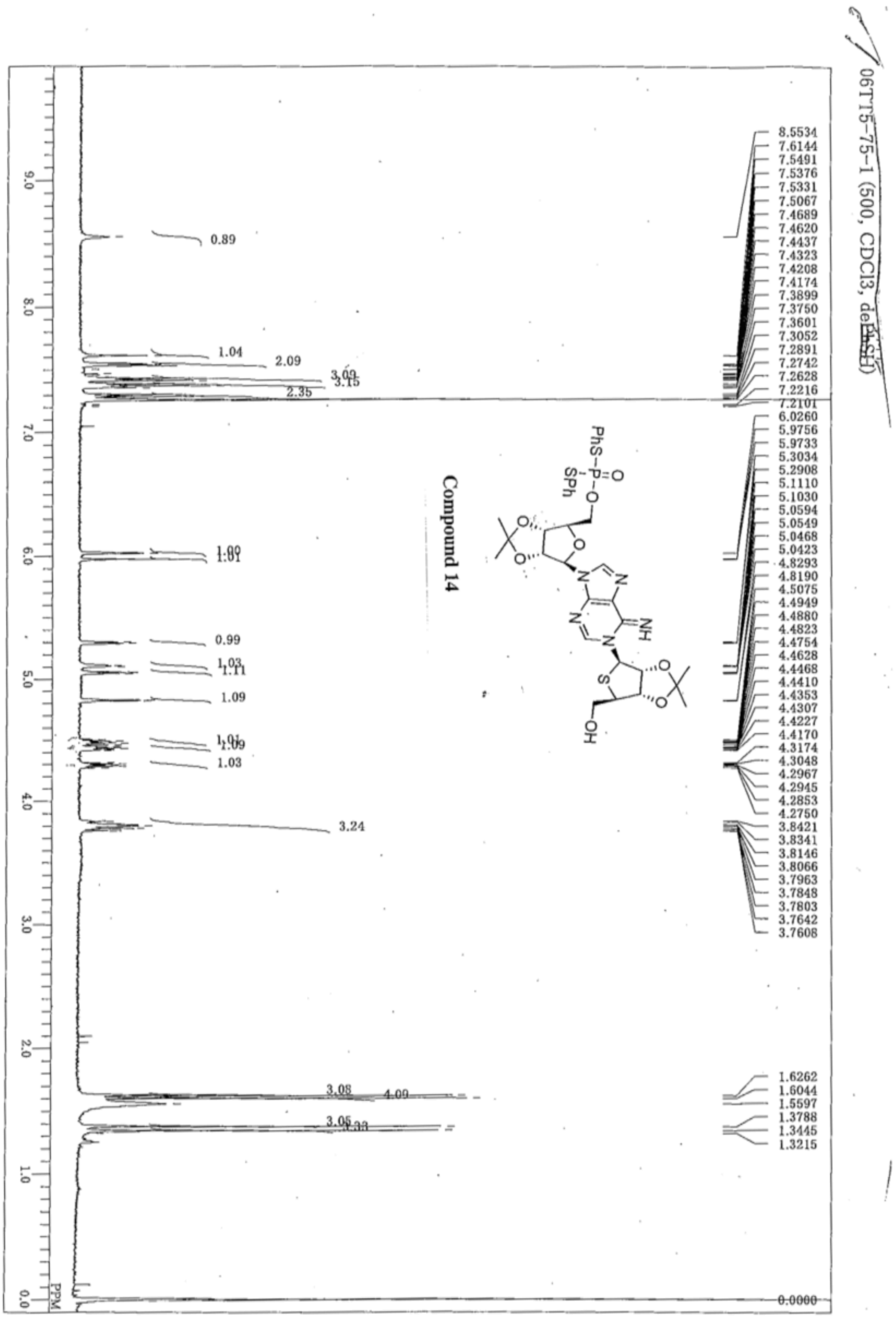
Compound 14
06T15-75-1 (500, CDCl3,

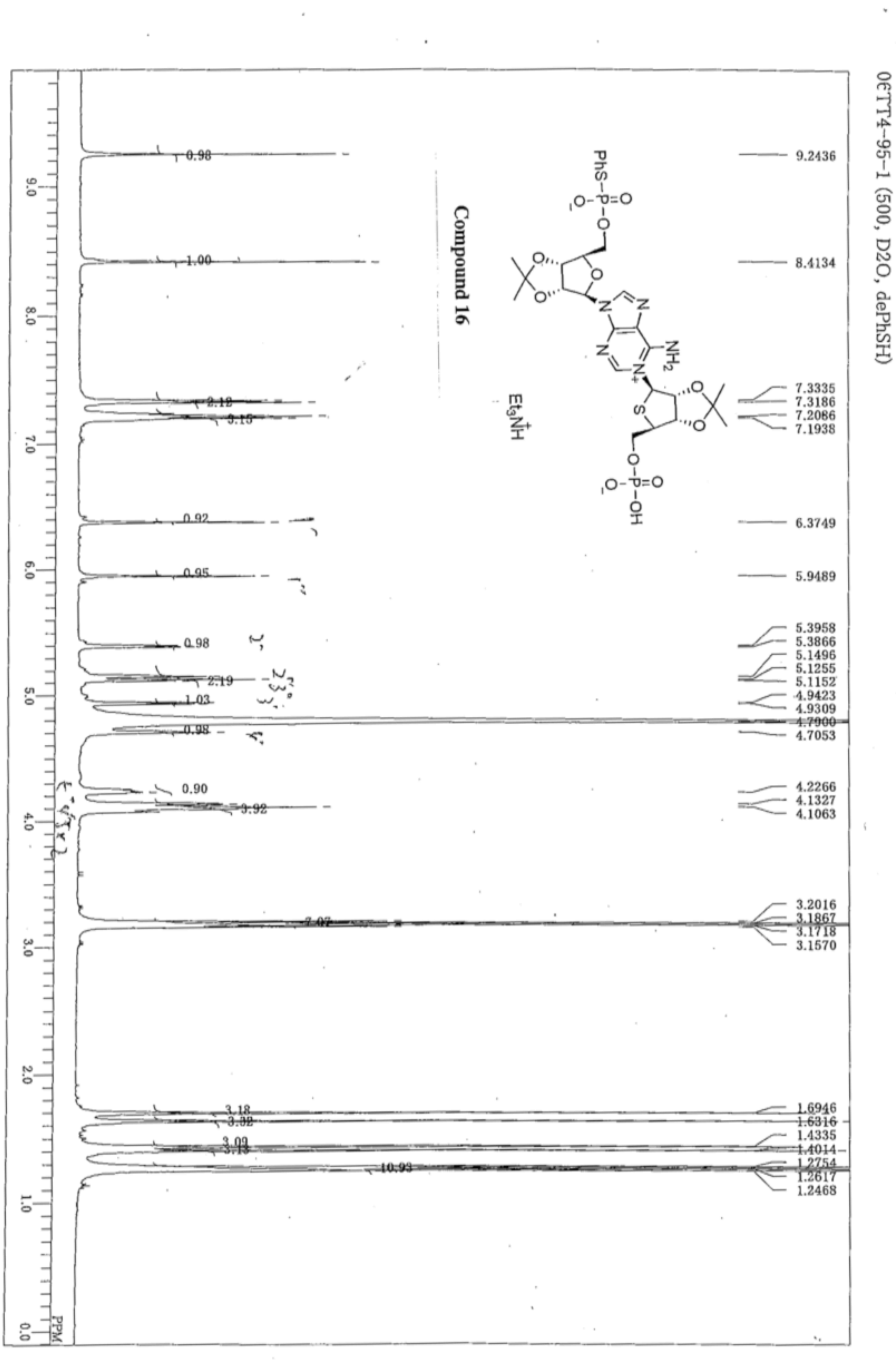
TT4-95-1 (500, D2O, dePhSH)
Compound 16
Et3NH+
9.2436
8.4134
7.3335
7.3186
7.2086
7.1938
6.3749
5.9489
5.3958
5.3866
5.1496
5.1255
5.1152
4.9423
4.9309
4.7900
4.7053
4.2266
4.1327
4.1063
3.2016
3.1867
3.1718
3.1570
1.6946
1.6316
1.4335
1.4014
1.2754
1.2617
1.2468
PPM

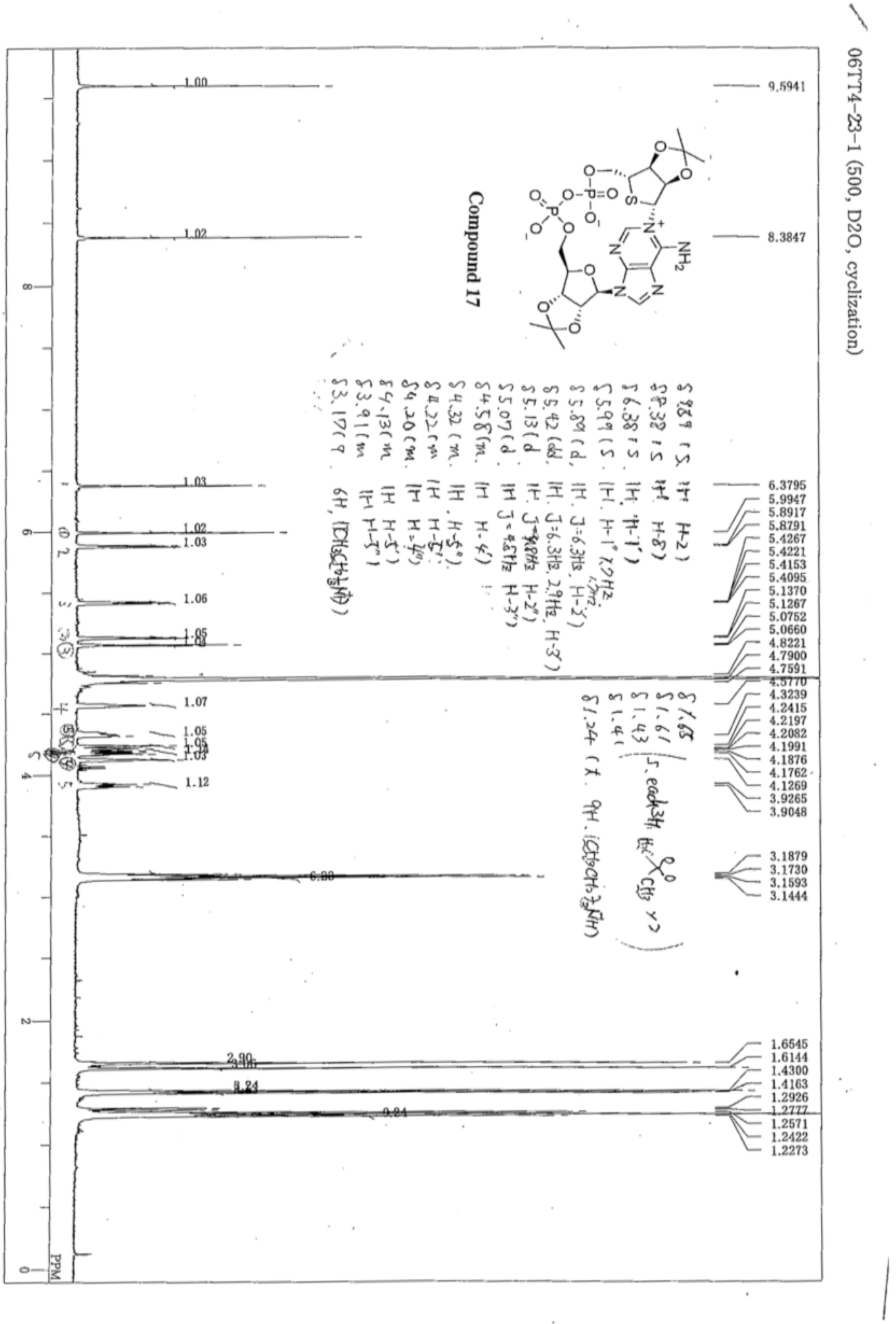
06TT4-23-1 (500, D2O, cyclization)
Compound 17
PPM
9.5941
8.3847
6.3795
5.9947
5.8917
5.8791
5.4267
5.4221
5.4153
5.4095
5.1370
5.1267
5.0752
5.0660
4.8221
4.7900
4.7591
4.5770
4.3239
4.2415
4.2197
4.2082
4.1991
4.1876
4.1762
4.1269
3.9265
3.9048
3.1879
3.1730
3.1593
3.1444
1.6545
1.6144
1.4300
1.4163
1.2926
1.2777
1.2571
1.2422
1.2273

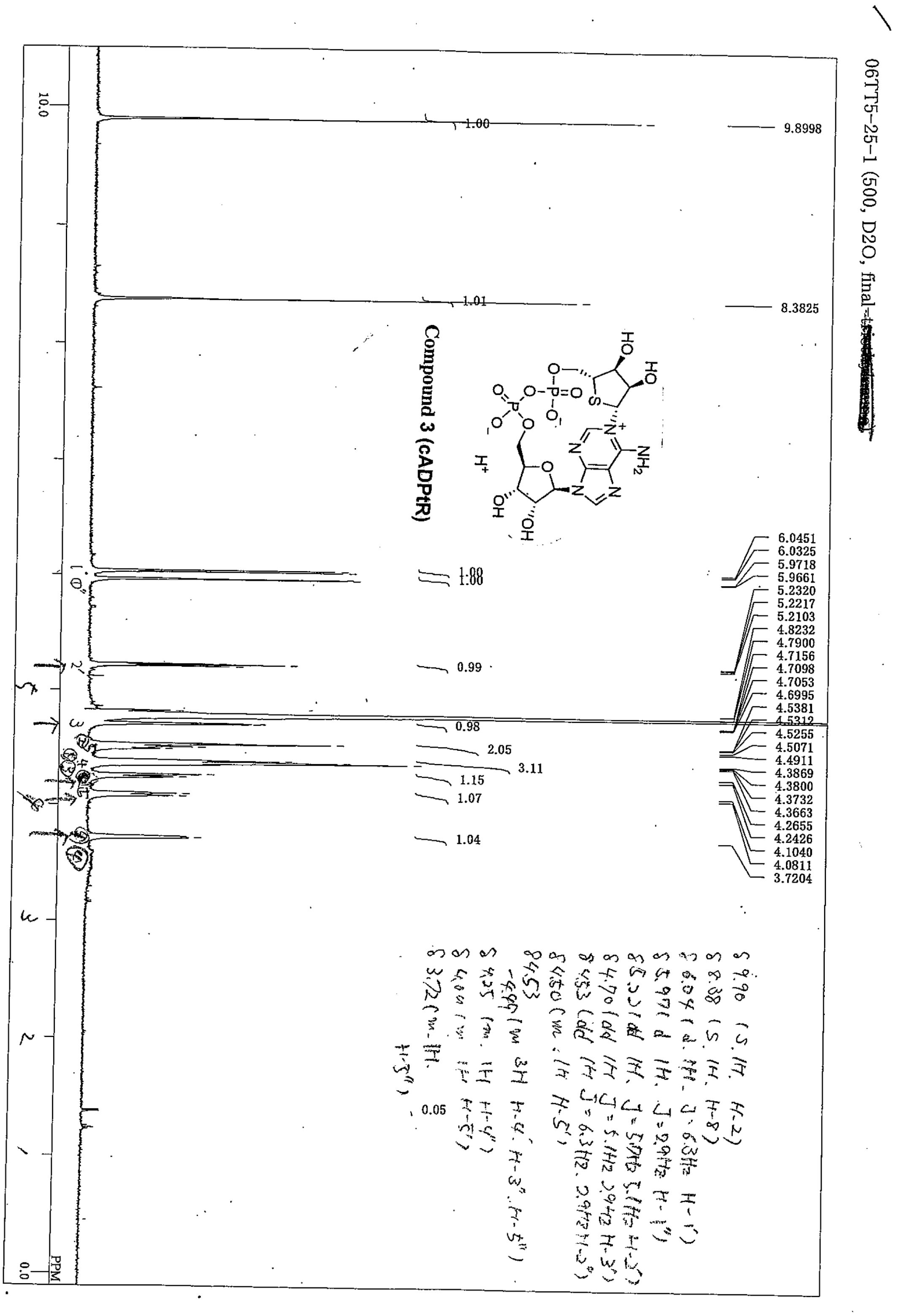
06T5-25-1 (500, D2O, final-
Compound 3 (cADPtR)
9.8998
8.3825
6.0451
6.0325
5.9718
5.9661
5.2320
5.2217
5.2103
4.8232
4.7900
4.7156
4.7098
4.7053
4.6995
4.5381
4.5312
4.5255
4.5071
4.4911
4.3869
4.3800
4.3732
4.3663
4.2655
4.2426
4.1040
4.0811
3.7204
1.00
1.01
1.08
0.99
0.98
2.05
3.11
1.15
1.07
1.04
0.05
10.0
0.0
PPM
